# CRISPR/Cas9-Mediated Immunity to Geminiviruses: Differential Interference and Evasion

**DOI:** 10.1038/srep26912

**Published:** 2016-05-26

**Authors:** Zahir Ali, Shakila Ali, Manal Tashkandi, Syed Shan-e-Ali Zaidi, Magdy M. Mahfouz

**Affiliations:** 1 Laboratory for Genome Engineering, Division of Biological Sciences, 4700 King Abdullah University of Science and Technology, Thuwal 23955-6900, Saudi Arabia

## Abstract

The CRISPR/Cas9 system has recently been used to confer molecular immunity against several eukaryotic viruses, including plant DNA geminiviruses. Here, we provide a detailed analysis of the efficiencies of targeting different coding and non-coding sequences in the genomes of multiple geminiviruses. Moreover, we analyze the ability of geminiviruses to evade the CRISPR/Cas9 machinery. Our results demonstrate that the CRISPR/Cas9 machinery can efficiently target coding and non-coding sequences and interfere with various geminiviruses. Furthermore, targeting the coding sequences of different geminiviruses resulted in the generation of viral variants capable of replication and systemic movement. By contrast, targeting the noncoding intergenic region sequences of geminiviruses resulted in interference, but with inefficient recovery of mutated viral variants, which thus limited the generation of variants capable of replication and movement. Taken together, our results indicate that targeting noncoding, intergenic sequences provides viral interference activity and significantly limits the generation of viral variants capable of replication and systemic infection, which is essential for developing durable resistance strategies for long-term virus control.

Geminiviruses threaten food security and agriculture, infecting key crop species, especially in tropical and subtropical regions^1^. Geminiviruses are characterized by their twin icosahedral capsids and small, single-stranded DNA (ssDNA) genome (∼2.7 kb)^2^. A study examining genome-wide pairwise sequence identity, genome organization, host range, and insect transmission vector recently classified the family *Geminiviridae* into seven genera: *Begomovirus*, *Curtovirus*, *Topocuvirus*, *Mastrevirus*, *Becurtovirus*, *Turncurtovirus*, and *Eragroviru*^3^. Begomoviruses infect dicotyledonous plants via the silverleaf whitefly (*Bemisia tabaci*) vector. The genomes of Begomoviruses are composed of one (A, monopartite) or two (A and B, bipartite) components. The A and B components of the virus share a common region with nearly identical nucleotide sequences^4^.

Effective strategies for controlling geminiviruses remain expensive and inefficient, due to mixed virus infections and the patho-interaction of vectors, viruses, and host plants^5^. However, recent work showed that site-specific nucleases can directly target and cleave the viral genome. This cleavage of the viral genome leads to the generation of double strand breaks (DSBs), which are either repaired by the imprecise non-homologous end-joining repair (NHEJ) machinery or by precise homology-directed repair (HDR)^6^,^7^,^8^. The presence of unrepaired DSBs ultimately leads to degradation of the virus molecules^9^. Virus variants generated by NHEJ can replicate and move systemically only if NHEJ maintains the proper “frame” for translation and does not compromise protein function. Several site-specific nuclease platforms have been developed with potential applications for targeted interference against viral genomes.

Clustered regularly interspaced palindromic repeats (CRISPR)/CRISPR-associated 9 (Cas9) is an adaptive molecular immunity system used by bacteria and *Archaea* to fend off invading phages and conjugative plasmids^10^,^11^,^12^. The CRISPR/Cas9 system has been harnessed for targeted mutagenesis and genome editing of eukaryotic genomes, including plants^13^,^14^,^15^,^16^,^17^. The CRISPR/Cas9 machinery is composed of Cas9 (a site-specific DNA endonuclease) and a synthetic single guide RNA (sgRNA). The sgRNA, which carries 20-nuclecotides of target sequence information, is used to direct the Cas9 endonuclease to its genomic target sequence, which must precede a tri-nucleotide sequence known as the protospacer-associated motif (PAM). *Streptomyces pyogenes* Cas9 recognizes the PAM sequence NGG and cleaves three nucleotides preceding this PAM sequence on complementary and non-complementary strands^10^,^11^,^12^,^15^,^16^. Cas9 variants with improved specificity have recently been characterized^18^,^19^,^20^,^21^.

We and other groups have recently demonstrated the portability of the CRISPR/Cas9 machinery to confer molecular immunity against eukaryotic viruses, including plant DNA viruses^7^,^22^,^23^,^24^,^25^,^26^. We demonstrated that the CRISPR/Cas9 machinery can target coding and non-coding sequences of different geminiviruses^7^,^27^,^28^. This targeting results in reduced viral accumulation and delayed or abolished symptoms. However, different sgRNAs have different targeting efficiencies for coding or noncoding sequences^7^. Numerous reports describe the emergence of geminiviruses with altered pathogenicity and subsequent changes in the severity of disease symptoms in infected plants, resulting from recombination-mediated genetic changes or reassortment among different viral genomes^2^. Targeting the viral genome opens up various possibilities, including degradation and/or repair of these genomes. Under natural field conditions, where mixed viral infections exist, targeting a single virus, generating DSBs, and initiating cellular repair could induce recombination or generation of viral variants capable of replication and survival. Since the nature of the target viruses largely determines the efficiency of interference by the CRISPR/Cas9 system, production of durable resistance requires the establishment of criteria for selecting which virus sequences to target.

In this study, we investigated the ability of the CRISPR/Cas9 machinery to interfere with different monopartite (CLCuKov) and bipartite viruses (MeMV) and different severe and mild strains of TYLCV geminiviruses. We found that sgRNAs designed to target coding and noncoding sequences targeted the respective sites in different viruses with different efficiencies. Furthermore, sgRNAs targeting the coding sequences of all viruses were effective for interference and for generating virus variants, but those targeting the non-coding intergenic regions (IRs) of CLCuKoV and TYLCSV were capable of interference, but inefficient in generating virus variants. We also investigated the replicative ability of mutated viral variants that could evade the CRISPR/Cas9 system.

Our results indicate that CRISPR/Cas9-induced variants in open reading frames (ORFs) of geminiviruses were capable of replication and systemic movement, thereby evading the CRISPR/Cas9 machinery, but the IR variants failed to replicate and move systemically. Our results provide important insights for developing effective strategies for CRISPR/Cas9-based molecular interference and will facilitate investigations of the basic biology of geminiviruses.

## Results

### The coding sequences of CLCuKoV are targeted for cleavage by CRISPR/Cas9 and subsequently repaired by NHEJ

**Figure 1:**
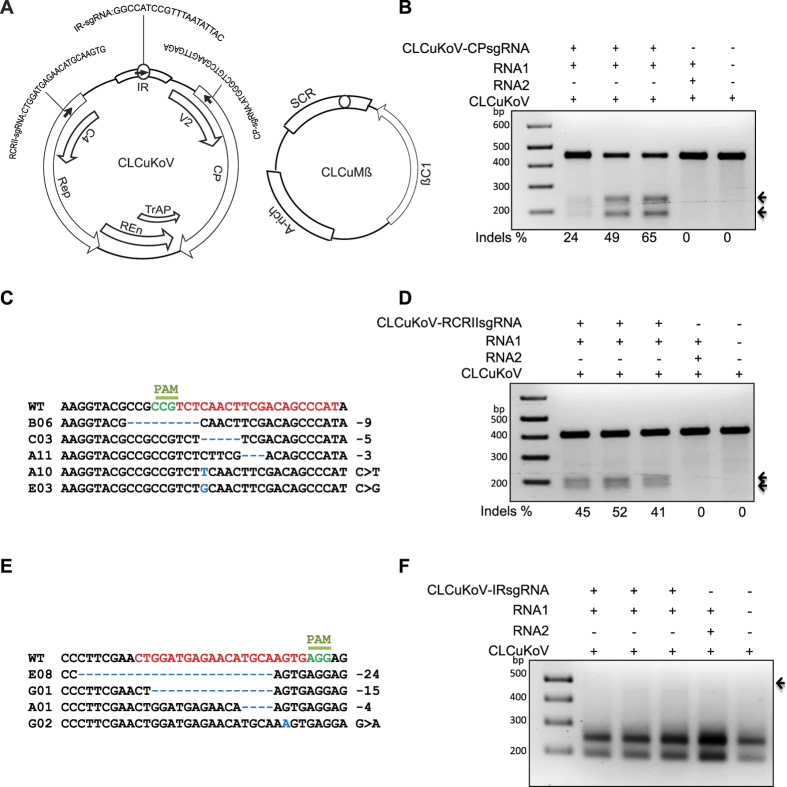
CRISPR/Cas9-mediated targeting of coding and non-coding sequences of the CLCuKoV genome. (**A**) Genome organization of CLCuKoV and CLCuMß. Bidirectional and overlapping ORFs (CP, Rep, Ren, TrAP, V2, and C4) are represented by arrows, the IR by a box, stem loop nonanucleotides by a small circle in the IR and SCR, and targets by arrowheads and individual sequences. The selected targets, one in non-coding IR, one each in coding CP or in the Rep RCRII domain, were analyzed for CRISPR/Cas9-mediated targeting (**B**) NHEJ repair (indel) analysis via the T7EI assay. Arrow indicates the presence of 255 bp and 191 bp regions only in samples expressing CP-sgRNA, but not in samples with TRV empty vector or virus alone. (**C**) Alignment of PCR amplicons encompassing the CP region and subjected to Sanger sequencing for indel (NHEJ repair) confirmation. (**D**) T7EI assay detecting indels in the RCRII domain of CLCuKoV genome. Mutations were detected only in RCRII PCR amplicons from plants infiltrated with TRV containing RCRII-sgRNA, but not in plants infiltrated with TRV empty vector or virus alone. (**E**) Alignment of PCR amplicons encompassing the RCRII motif and subjected to Sanger sequencing for NHEJ repair confirmation. (**F**) NHEJ repair analysis at the IR sequence by restriction site loss assay. The arrow indicates the expected *Ssp*I-resistant 446 bp DNA fragment; none of the samples produced the *Ssp*I-resistant DNA fragment, which is similar to TRV empty vector or virus alone. Arrows in (**A**), (**C**) and (**E**) represent the expected DNA fragments. The indel percentage shown was calculated based on the Sanger sequence reads. In (**B**) (reverse strand sequence) and (**D**) the wild-type (WT) sequences are shown at the top (target sequence is shown in red; the protospacer-associated motif [PAM] in green, followed by the various indels formed, as indicated by numbers to the right of the sequence (−, deletion of x nucleotides; + , insertion of x nucleotides; and > , change of x nucleotides to y nucleotides). CP coat protein, Rep replicase, Ren replication enhancer, TrAP transcriptional activator protein, βC1 betasatellite conserved ORF, IR intergenic region, SCR satellite conserved region.

We recently demonstrated the use of the CRISPR/Cas9 system to confer interference activities against monopartite plant DNA viruses^7^. In this study, we explored the utility of the CRISPR/Cas9 system to confer molecular immunity against complex plant DNA viruses, i.e., viruses having partial or complete partite genomes. Therefore, we selected the monopartite virus *Cotton Leaf Curl Kokhran Virus* (CLCuKoV; AJ496286), which requires the helper betasatellite Cotton Leaf Curl Multan Betasatallite (CLCuMß) for symptom development^29^. To deliver sgRNAs specific to the coding regions of the genes encoding the coat protein (CP) and the divalent cation coordination RCRII domain of replication associated protein (Rep) (Fig. 1A) of CLCuKoV, the sgRNAs were delivered through *Tobacco rattle virus* (TRV) RNA2 into Cas9OE *Nicotiana benthamiana* plants^30^. Next, the plants with an established sgRNA-Cas9 complex were challenged *via* agro-infiltration with infectious clones of CLCuKoV and CLCuMß^29^,^31^. Next, we isolated total genomic DNA from systemic leaves to investigate whether CRISPR/Cas9 can target and cleave the CLCuKoV genome and whether the DSBs generated by the CRISPR/Cas9 system would be repaired via NHEJ. We subjected the PCR amplicons encompassing the targets to T7 Endonuclease I (T7EI) mutation detection analysis, where denatured DNAs are re-natured, then cleaved with T7EI, which degrades single-stranded regions of non-complementarity resulting from indels; these regions are then detected by sequencing. High rates of indel formation were detected at both ORFs (18–49% in CP and 35–45% in RCRII) of CLCuKoV (Fig. 1B,D). Subsequently, we cloned the PCR amplicons into the pJet2.1 cloning vector and subjected them to Sanger sequencing to identify the nature of the modifications in the CLCuKoV genome. The results confirmed the presence of indels and indicated that Cas9 activity was high and was followed by NHEJ repair. All possible types of indels were observed (Fig. 1C,E).

To investigate whether CRISPR/Cas9 can target the well-conserved IR of CLCuKoV and whether cleavage results in the same rate of NHEJ repair, we assayed for loss of a restriction enzyme recognition site. The invariant 9-nt conserved sequence in the IR contains a recognition site for *Ssp*I endonuclease and is located at the predicted Cas9 cleavage site, 3 bp upstream of the PAM sequence. The flanking 446-bp fragment encompassing the target sequence (Supplementary Sequence 1) was digested with *SspI*. Complete *Ssp*I digestion of the wild-type (WT) sequence produced two fragments of 253 and 193 bp. Cas9 targeting and subsequent repair via NHEJ should eliminate the *Ssp*I site within the conserved 9-nt sequence and thus should generate a 446 bp fragment of *Ssp*I-resistant DNA. Surprisingly, we did not detect an *SspI*-resistant fragment in an agarose-based gel assay (Fig. 1F). Previously, we showed that multiple viruses can be targeted by a single sgRNA with an invariant (TAATATTAC) sequence common to all geminiviruses^7^,^32^. Next, we investigated whether CLCuKoV could be targeted by an invariant sgRNA. We used different sgRNAs specific to the IRs of monopartite BCTV and TYLCV or bipartite MeMV against CLCuKoV. A restriction enzyme site loss assay did not reveal a significant level of indel formation (Supplementary Figure 1).

### CRISPR/Cas9-mediated, efficient targeting of coding and non-coding sequences of MeMV

**Figure 2:**
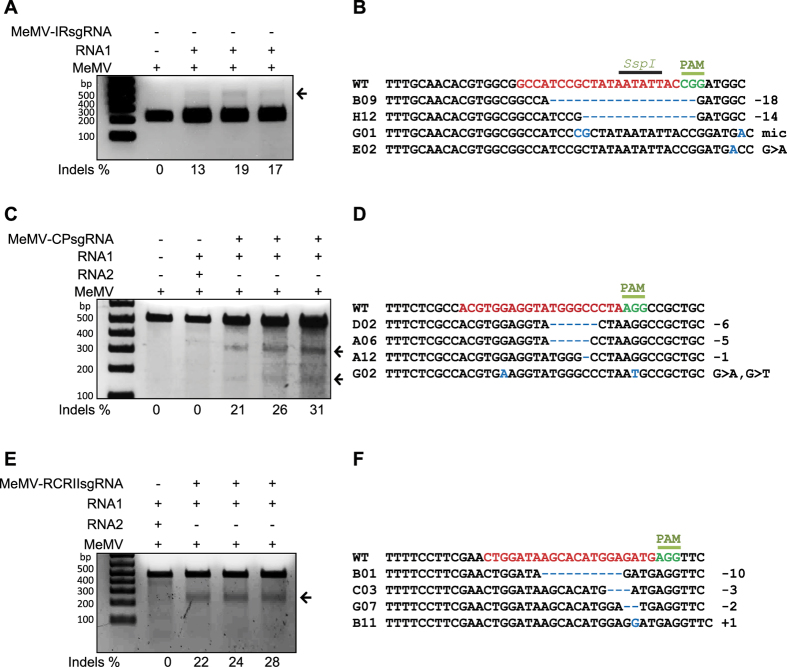
NHEJ repair of coding and non-coding sequences of the MeMV genome. Non-coding IR, coding CP, and the Rep RCRII domain of MeMV were analyzed for NHEJ repair. (**A**) NHEJ repair (indel) analysis via an *SspI* recognition site loss assay. The MeMV IR (453 bp) was analyzed for the loss of the *Ssp*I recognition site at the CRISPR/Cas9 target locus. Unlike CLCuKoV, NHEJ-repaired indels are indicated by arrows pointing to the 453-bp *Ssp*I-resistant DNA fragments. (**B**) Alignment of reads of PCR amplicons encompassing the IR of MeMV subjected to Sanger sequencing for NHEJ repair confirmation. (**C**) T7EI assay to detect indels in the CP of the MeMV genome. The T7EI assay detected indels only in CP PCR amplicons from plants infiltrated with TRV containing the CP-sgRNA, but not in plants infiltrated with TRV empty vector or virus alone. (**D**) Alignment of reads of the PCR amplicons encompassing the target site and subjected to Sanger sequencing for NHEJ repair confirmation. (**E**) NHEJ repair analysis at the RCRII motif of MeMV by T7E1 assay. Arrow indicates the expected DNA fragments; TRV empty vector or virus alone did not show similar fragments. All DNA fragments from (**A,C and E**) were resolved on an 2% agarose gel premixed with ethidium bromide stain. Arrows in (**A,C and E**) indicate the expected DNA fragments. The indel percentage shown below each gel was calculated based on the Sanger sequence reads. In (**B,D and F**), the wild-type (WT) sequences are shown at the top (target sequence is shown in red; the protospacer-associated motif [PAM] is indicated in green, followed by the various indels formed, as indicated by numbers to the right of the sequence (−, deletion of x nucleotides; +, insertion of x nucleotides; and >, change of x nucleotides to y nucleotides).

Next, we investigated whether the lack of NHEJ repair in the IR after CRISPR/Cas9 targeting is common in all partial or full bipartite viruses. We selected the bipartite virus MeMV with genomic parts DNA-A and DNA-B (Supplementary Figure 2, Supplementary Sequence 2). We systemically delivered sgRNA specific to the non-coding IR into plants. The NB-Cas9OE plants were subsequently challenged via agro-infection with MeMV DNA-A and DNA-B. At 15 days post infection (dpi), PCR amplicons encompassing the IR target site were examined using the *SspI* enzyme loss assay. The *SspI* resistance results confirmed that, in contrast to CLCuKoV, CRISPR/Cas9 cleaved the stem loop sequence in the IR of MeMV, which was subsequently repaired by NHEJ (Fig. 2A). Sanger sequencing revealed the identity of the indel modification. In contrast to CLCuKoV, 13–19% of MeMV IRs were repaired by NHEJ, and all normal types of indels were produced (Fig. 2B). Notably, other types of modifications were also observed, albeit at low frequency, including long deletions (Supplementary Figure 3). Consistent with this, multiple variants of IR-sgRNA targeted MeMV-A IR sequences with varying efficiencies (12–30%) (Supplementary Figure 4). As MeMV DNA-B has the same 20-nt sequence and can be targeted by the same sgRNA, our restriction site loss assay confirmed that, like MeMV DNA-A, MeMV DNA-B is targeted and repaired through NHEJ (Supplementary Figure 5). Like the non-coding IR, targeting of the ORF sequences CP and RCRII of MeMV DNA-A produced high rates of indel formation (Fig. 2C,E). However, in contrast to indels at the IR, the CP and RCRII regions of MeMV-A are only short indels produced by NHEJ, as we did not observe any long deletions at the targeted sites (Fig. 2D,F).

### Variable efficiencies of indel formation at the IR sequences of different strains of TYLCV

**Figure 3:**
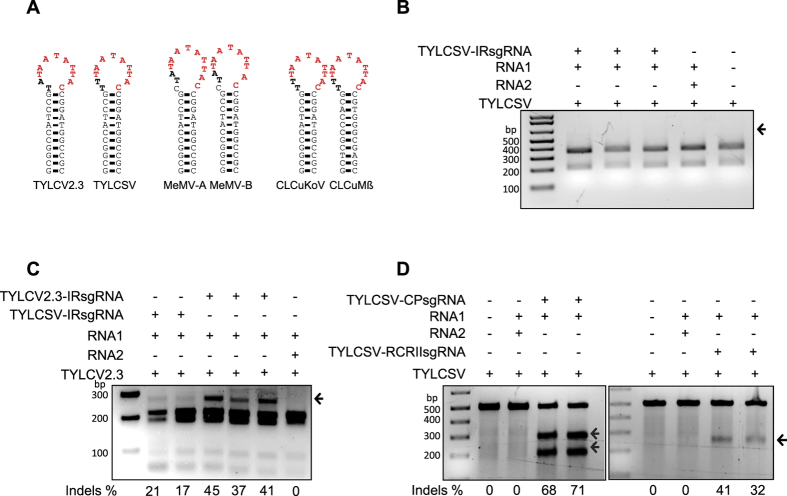
Variable efficiencies of indel formation at the IR sequences of different strains of TYLCV. (**A**) Stem-loop structure of the different geminiviruses used in this study. The conserved nonanucleotide motif is flanked on each side by a short stretch of complementary sequences. (**B**) Restriction site loss assay for detecting NHEJ-based indels at the IR of TYLCSV. The TYLCSV IR (562 bp) was analyzed for the loss of the *Ssp*I recognition site at the targeting locus. The arrow indicates the location of the expected 562-bp *Ssp*I-resistant DNA fragment in samples with IR-sgRNA, but like TRV empty vector and virus alone, no SspI-resistant fragment was observed. (**C**) *SspI* recognition site loss assay for detecting indels at the IR in TYLCV2.3. The variant TYLCSV-IRsgRNA (two lanes after marker) and authentic TYLCV2.3-IRsgRNA (last three lanes) were used to target the IR of TYLCV2.3. Arrows indicate the presence of the expected 269 bp *Ssp*I-resistant DNA fragments in samples with TYLCV2.3-IR-sgRNA or TYLCSV-IRsgRNA, but no *SspI*-resistant fragment was observed in TRV empty vector. (**D**) T7EI assay for detecting NHEJ-based indels in the CP and RCRII domain of the TYLCSV genome. T7EI assay detected high rates of indel formation both in CP and RCRII PCR amplicons from plants infiltrated with TRV containing CP or RCRII-sgRNA compared with TRV empty vector. DNA fragments of (**B,C and D**) were resolved on a 2% agarose gel stained with ethidium bromide. Arrows represent the expected DNA fragment in T7EI-digested DNA. The indel percentage shown below each gel was calculated based on the Sanger sequence reads.

In geminiviruses, the highly conserved nonanucleotide sequence is flanked by a stretch of short, complementary sequences, which form a stem-loop structure in the IR (Fig. 3A and Supplementary Figure 6). This conserved structure is directly involved in virus replication as a site for Rep binding and nick formation, and has putative bidirectional promoters^4^,^33^,^34^. Also, IRs are strain-specific and interact only with specific Rep proteins^35^,^36^,^37^. The presence of variable targeting and indel formation at the IR sequences of CLCuKoV and MeMV prompted us to investigate whether this would be the case in different strains of TYLCV. Therefore, we used the authentic TYLCV2.3-IR-sgRNA to target TYLCV2.3 and the TYLCSV-IR-sgRNA to target TYLCSV^38^. *N. benthamiana* plants overexpressing the CRISPR/Cas9 machinery were challenged with infectious clones of TYLCV2.3 and TYLCSV via agro-infection. At 15 dpi, we PCR amplified the fragments encompassing the IR target sequences and subjected the PCR amplicons to an *SspI* recognition site loss assay, which confirmed the absence of detectable levels of indels (Fig. 3B).

To exclude the possibility that the inability to detect any indels was due to a factor other than the inability of the CRISPR/Cas9 machinery to target the viral genome and to generate DSBs, we used the same machinery with the TYLCSV-sgRNA to target TYLCV2.3 and detected indel formation. The *SspI* recognition site loss assay confirmed that, like TYLCV2.3-IRsgRNA, TYLCSV-IRsgRNA could target the TYLCV2.3 genome, resulting in indel formation at the IR site of TYLCV (Fig. 3C). Moreover, no indels were detected after targeting TYLCSV IR with invariant sgRNAs (Supplementary Figure 7). However, in contrast to the inability to detect indels at IRs of TYLCSV, higher frequencies of indel formation were observed in the ORFs encoding CP and the RCRII domain of Rep (71% for CP and 41% for RCRII) (Fig. 3D). These indels were produced through NHEJ repair (Supplementary Figure 8A,B).

### CRISPR/Cas9 targets the noncoding IRs of CLCuKoV and TYLCSV

**Figure 4:**
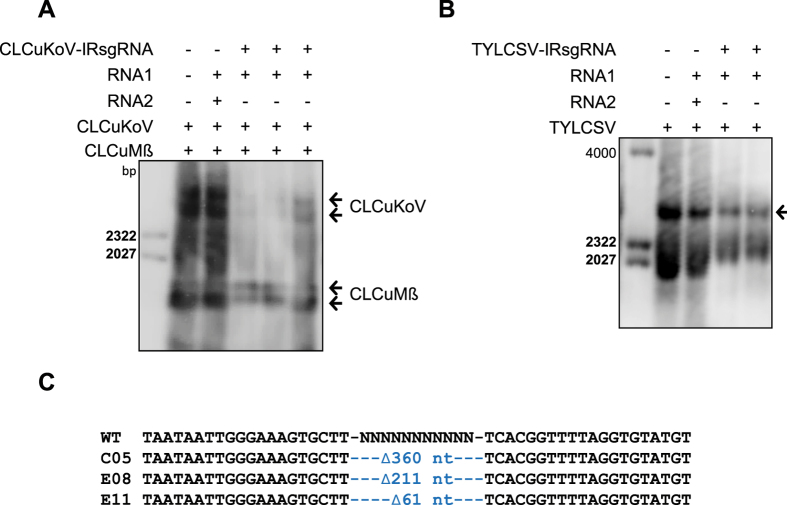
IR targeting by CRISPR/Cas9 interferes with genome accumulation of both CLCuKoV and TYLCSV. (**A**) DNA blot analysis assaying CLCuKoV (**A**) and TYLCSV (**B**) genomic DNA accumulation in Cas9OE plants expressing CLCuKoV-IRsgRNA and TYLCSV-IRsgRNA, respectively. CLCuKoV and TYLCSV genomic DNA was detected with DIG-labeled probe produced against the respective IRs of CLCuKoV and TYLCSV. All individual plants with IR-sgRNA that were infiltrated with CLCuKoV and TYLCSV exhibited reduced accumulation of the genomes relative to plants inoculated with TRV empty vector and virus only. Arrowheads in (**A,B**) indicate detection of the expected size of the TYLCV genome. (**C**) Alignment of cloned Sanger-sequenced PCR amplicons encompassing the IR of CLCuKoV. Alignment of sequence reads encompassing the IR shows only long deletions. The wild-type (WT) CLCuKoV sequence is shown at the top; the various indels formed are indicated by numbers in the middle of the sequence reads.

To investigate the possibility that the CRISPR/Cas9 machinery targeted the CLCuKoV and TYLCSV genomes but that these genomes were neither repaired nor proficient in replication, we used DNA blot analysis to examine the total DNA from infected plants for the accumulation of CLCuKoV and TYLCSV genomic DNA. The results confirmed that compared to control plants, plants with the CRISPR/Cas9 machinery targeting the IR sequence accumulated lower levels of the CLCuKoV (Fig. 4A) and TYLCSV genomes (Fig. 4B). The probe used to detect CLCuKoV also detected CLCuMß, but the amount of CLCuMß did not decrease as much as did CLCuKoV, which is consistent with the observation that the 20-nt target sequence in the IR (including the conserved nonanucleotide) of CLCuMß is not followed by a PAM (NGG) (Supplementary sequence 1). Nonetheless, we did not detect indels in an agarose gel-based *SspI* site loss assay. Sanger sequencing of CLCuKoV IR, either targeted by its authentic IR-sgRNA or by an invariant IR-sgRNA, confirmed that CRISPR/Cas9 targeted the IR of the CLCuKoV genome, but the level of repair was extremely low (3%). Furthermore, we observed the presence of only long deletions and a lack of the usual short indels (Fig. 4C and Supplementary Figure 9). Similarly, the results of sequencing indicated that targeting CLCuKoV with an invariant sgRNA occurred at a similar, low level (3%), and all targeted regions had long deletions, like those generated by the authentic IR-sgRNA against the CLCuKoV genome (Supplementary Figure 10).

### NHEJ repair of the coding regions of geminivirus genomes facilitates the generation of protein variants and evasion of the CRISPR/Cas9 system

**Figure 5:**
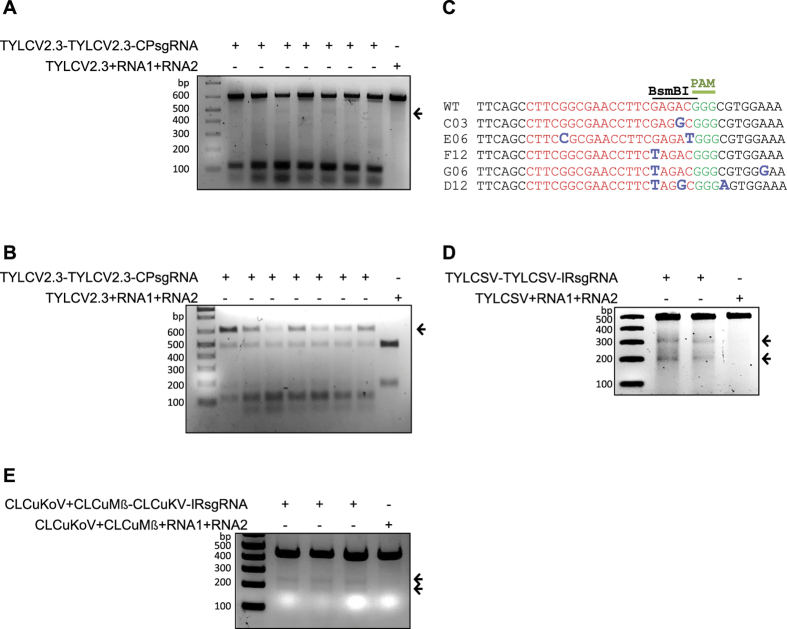
NHEJ-repaired CP sequence evades CRISPR/Cas9. (**A**) Evasion of repaired CP sequence of TYLCV genomes, as revealed by the T7EI assay. Sap from TYLCV-infected plants with an established CRISPR/Cas9 system against the CP region was applied to WT *N. benthamiana* plants. Total genomic DNA was isolated from top (young) leaves of sap-inoculated WT plants at 15 DAI. CP targets flanking PCR amplicons were subjected to T7EI. Arrows indicate the presence of the expected digested DNA fragment from samples of CP-targeted sap-infected plants compared to TRV empty vector with TYLCV. (**B**) *BsmBI*-recognition site loss assay for detecting escapees. *BsmBI*-treated PCR fragments were used as template in another round of PCR with the same primers. Purified DNA from this PCR was again subjected to *BsmBI* digestion. Arrowheads indicate the expected *BsmBI*-resistant DNA fragments compared to WT PCR amplicons from TRV empty vector. (**C**) Alignment of Sanger-sequencing reads of PCR amplicons encompassing the CP region of TYLCVfor mutation at the CRISPR/Cas9 targeting site. The wild-type (WT) TYLCV sequences are shown at the top (target sequence is shown in red, the *BsmBI* site by a line, and the protospacer-associated motif [PAM] is indicated in green; the various indels formed are shown in enlarged, bold, and blue font at their respective sites. (**D**) Evasion analysis of TYLCSV genomes with repaired CP sequences via the T7EI assay. For TYLCSV samples were prepared as in (**A**) CP targets flanking the PCR amplicons were subjected to T7EI. Arrows indicate the presence of expected digested DNA fragments from samples of CP-targeted sap-infected plants compared to TRV empty vector with TYLCSV. (**E**) Evasion analysis of genomes of CLCuKoV genomes with repaired CP sequences via the T7EI assay. Wild-type scions were grafted to the stocks of CLCuKoV-infected plants with an established CRISPR/Cas9 system against the CP region. Total genomic DNA was isolated from top (young) leaves of WT scions at 21 DAI. CP-target-flanking PCR amplicons were subjected to T7EI. Arrows indicate the presence of the expected digested DNA fragment from samples of CP-targeted CLCuKoV compared to TRV empty vector with CLCuKoV.

We observed high rates of indels formation in the genomes of different geminiviruses after targeting the protein coding region or the non-coding regions (IRs) of TYLCV2.3 and MeMV. We therefore investigated: 1) whether indels created at these sites might allow the virus to evade the CRISPR/Cas9 machinery and if these viral variants would be considered escapees and 2) whether these escapees can replicate and produce a systemic infection. To address these questions, the sap from infected, CRISPR/Cas9-targeted plants (CP and IR of TYLCV2.3) was rub-inoculated onto leaves and directly injected into the leaf pedicels and stems of three-week-old WT *N. benthamiana* plants (Supplementary Figure 11). Samples were collected from the systemic top (young) leaves at 15 DAI. The respective CP and IR fragments were PCR amplified and subjected to T7EI and restriction site loss assays to detect mutations. The results indicate that some of the CP sequences repaired by NHEJ were capable of replication and systemic spreading in WT *N. benthamiana* (Fig. 5A and B). To confirm our T7EI and restriction site loss assay results, we used Sanger sequencing to determine the identity of these indels. The results revealed the presence of indels in the target sequences of the viral genomes, indicating that they were escapee variants capable of replication (Fig. 5C). By contrast, we did not detect any indels (escapees) in the IR samples (Supplementary Figure 12). To confirm that targeting a protein coding sequence of other geminiviruses could lead to similar CRISPR/Cas9 escapees, we performed experiments using TYLCSV and CLCuKoV. For TYLCSV, we employed the same sap inoculation method used for TYLCV2.3, but for CLCuKoV, we used a grafting method with scions from WT plants grafted onto CRISPR/Cas9 CLCuKoV plants (Supplementary Figure 11). Subsequently, total DNA was extracted at 21 dpi and PCR amplicons encompassing the target sequences were subjected to T7EI assays. The T7EI results confirm the presence of the modified (CP) genomes in samples from both CLCuKoV (Fig. 5D) and TYLCSV (Fig. 5E). The modified indels in the CP of CLCuKoV were also confirmed by Sanger sequencing of the PCR-amplified fragments (Supplementary Figure 13). Furthermore, unlike the CP, we did not observe a mutated IR in either the T7EI or restriction site loss assay. Because targeting of the coding regions could lead to all kinds of mutations, we investigated these sequence modifications at the protein level by converting the Sanger sequencing reads to their respective amino-acids. As expected our results confirmed the presence of all types of mutations including nonsense and missense in both TYLCSV and CLCuKoV (Supplementary Figure 14A,B).

Finally, we investigated whether the rare genomic molecules with large deletions in the IR would be capable of replication or systemic spreading. These IR molecules were not detected in systemic leaves, indicating that they were unable to replicate or that they replicated at levels too low to be detected.

## Discussion

In this study, we investigated the efficiency of the CRISPR/Cas9 machinery for targeting different coding and non-coding sequences of geminivirus genomes and assessed the rate of indels formation in coding and non-coding regions of these viruses. We also examined whether virus variants were generated and whether the generation of these variants would eventually enable the virus to evade the CRISPR/Cas9 machinery. To investigate the versatility of the CRISPR/Cas9 system in conferring interference against different geminiviruses, we investigated the interference activity against CLCuKoV, which requires the helper betasatellite CLCuMß for disease symptom development. We tested sgRNAs targeting the coding and non-coding regions of CLCuKoV individually to determine their efficacy in directing the Cas9 endonuclease to the viral genome and establishing interference. Our data show that sgRNAs targeting the coding regions of the CP and RCRIII ORFs mediated high levels of interference, as evidenced by the indel mutation detection assays, which was subsequently confirmed by Sanger sequencing. By contrast, we did not detect indel modifications in the IR via the T7EI and restriction site loss assays using an sgRNA targeting the IR of the CLCuKoV or variant sgRNAs. These results suggest that either the sgRNA was inactive and incapable of directing the Cas9 endonuclease to the target virus sequence or that the targeting occurred but the cleaved virus sequences were subsequently degraded or the repaired variants were incapable of replication.

Next, we examined whether coding and non-coding sequences of other bipartite viruses could be targeted by CRISPR/Cas9, with subsequent NHEJ resulting in indel modifications. We targeted the bipartite MeMV (DNA-A and DNA-B genomes) and designed sgRNAs targeting the IR and ORFs (CP and RCRII, respectively). We found that sgRNAs targeting the coding and noncoding sequences were capable of targeting and interfering with subsequent NHEJ repair, as revealed by the T7EI and restriction site loss assays and Sanger sequencing. Therefore, the CRISPR/Cas9 system can interfere with bipartite viruses (MeMV) and target the IR, with subsequent NHEJ repair. Notably, differential CRISPR/Cas9-based indel formation in human viruses was also recently reported^27^,^28^,^39^.

Since we observed variable efficiencies of targeting the IRs of MeMV and CLCuKoV, we attempted to determine whether targeting the IRs of different strains of the same geminivirus would be similar or would vary. To investigate this, we used two different strains of TYLCV, i.e., TYLCV2.3 and the severe strain TYLCSV. Examination of the designed sgRNAs, including the invariant conserved nonanucleotide target sequence, revealed the absence of indels at the IR of TYLCSV. However, the same sgRNA, as well as another authentic sgRNA targeting the TYLCV2.3 IR, were able to target TYLCV2.3, and the IR was subsequently repaired by NHEJ, as evidenced by the presence of indel modifications in the T7EI and restriction site loss assays. Consistent with our previous results, all sgRNAs targeting the ORF (CP and RCRII) sequences were highly efficient in targeting all virus strains, with subsequent repair via NHEJ, as evidenced by the T7EI assays and Sanger sequencing.

The inability to detect any indel modifications in viral genomes from plants carrying sgRNAs targeting the IRs of TYLCSV and CLCuKoV prompted further investigation. Such inability could involve: 1) the inability of the sgRNA to target the Cas9 protein to the IR sequences of CLCuKoV and TYLCSV; 2) the inability of the NHEJ repair machinery to repair DSBs, ultimately leading to degradation of the cleaved IR sequences of these two viruses; or 3) the IR repaired variants of individual genomes were unable to replicate and were ultimately degraded. To determine which of these possibilities is in play, we investigated the interference activities by measuring the accumulation of the genomes of these two viruses. Interestingly, the sgRNAs targeting the IRs of CLCuKoV and TYLCSV were capable of directing the CRISPR/Cas9 machinery to the IR, thereby interfering with viral replication, ultimately resulting in a significant reduction in viral genome levels. Therefore, our findings indicate that the IR sgRNA can target the viral genome, but the subsequent repair of the DSBs via NHEJ is likely to generate viral variants inefficient in replication. This prompted us to sequence numerous amplicons to determine whether indel production or other types of modifications could have taken place. Sanger sequencing of the IR amplicons revealed the presence of a few IR sequences with large deletions ranging from 61–360 bp, indicating that these variants are inefficient in replication.

Our data reveal that the targeting and subsequent modification of coding regions via NHEJ repair are quite efficient and universal among the geminiviruses examined. However, sgRNAs targeting the IR are capable of interference, but the subsequent repair process results in variants inefficient in replication. Moreover, it is likely that amplicons with long deletions would be incapable of replication and systemic infection. Since we observed efficient NHEJ repair in the coding sequences of the CP and RCRII, we investigated whether these repaired sequences with indels, which constitute different protein variants, can replicate and survive, and could therefore move systemically throughout the plant. We investigated this hypothesis by applying CRISPR/Cas9 machinery targeting the coding sequences of the virus. Subsequently, we confirmed the presence of indels by mutation detection assays, and we used sap as a source of the virus variants generated by NHEJ repair to inoculate WT *N. benthamiana* plants. We then assayed for the presence of these variants in the systemic leaves of WT *N. benthamiana* plants. Virus variants were detected in the systemic leaves of WT *N. benthamiana* plants, indicating the capability of these variants to replicate and systemically move throughout the plant. By contrast, a similar set of experiments was performed using the sap from CRISPR/Cas9 machinery targeted to the IR. We did not detect any virus variants with modifications in the IR, corroborating our previous results and indicating that the NHEJ repaired variants are inefficient in replication. Notably, the ability of virus variants to replicate and subsequently dodge CRISPR/Cas9 was recently observed in HIV-1^39^.

Our findings provide important insights into the potential use of the CRISPR/Cas9 system for virus interference. Since NHEJ repair of the coding regions of different geminiviruses is quite efficient, leading to the generation of different viral variants capable of replication and systemic infection, it may be possible to generate viral variants that escape the CRISPR/Cas9 machinery. The selection and replication of these repaired variants would favor the more fit variants for virus survival. Since these viral variants could have variable replication efficiencies, it is expected that the variants with more proficient replication would predominate over those with less proficient replication. It also remains to be determined whether targeting certain genomic regions would increase the recombination frequencies under natural field conditions where mixed infections predominate.

The short genomes of viruses contain highly specialized, specific sequences required for specific functions including viral replication, evading host defense systems, and host infection. When designing interference strategies against viruses and coping with putative viral escape, the main focus is on the sequences that provide better interference when used for targeted interference^40^,^41^. Similarly, given the considerable heterogeneity and recombination ability of geminiviruses and the ability of CRISPR/Cas9-induced variants for replication and systemic movement, it would be conceivable to devise strategies where the CRISPR/Cas9 system targets the noncoding region (three nucleotides before the PAM) to inhibit or prevent the replication of the virus by mutating the essential parts of geminiviruses. Alternatively, targeting multiple regions of the virus genome simultaneously would abrogate the ability of the virus to use the NHEJ repair system, and these cleaved molecules would ultimately be degraded. Because the IR is critical for binding and replication initiation^42^, targeting this region by a catalytically inactive Cas9 would be expected to abrogate viral replication and subsequently lead to virus interference. In conclusion, our study highlights important aspects to consider when developing CRISPR/Cas9-based strategies for durable virus interference and resistance, thereby improving agricultural productivity.

## Methods

### Vector construction

A PCR-based restriction ligation procedure was used to clone the sgRNAs, as previously described^30^. Briefly, sgRNAs targeting the TYLCSV, MeMV-A, MeMV-B, and CLCuKoV genomes were cloned into the TRV RNA2 vector. A fragment containing the 20-nt target sequence, the 84-bp Cas9 binding loop for sgRNA, and a seven-T repeat (as a terminator) was amplified by PCR using a forward primer containing an *Xba*I recognition site, 20-nt target sequence, and 23-nt Cas9-binding sgRNA scaffold and a reverse primer containing an *Xma*I recognition site to amplify a 116-bp PCR fragment. All primer sequences used in this study are provided in supplementary information (Table 1). The 116-bp sgRNA for each target was cloned into the TRV RNA2 vector under the control of the PEBV promoter using restriction enzymes *Xba*I and *Xma*I. Sanger sequencing was used to confirm the sequences of all clones.

### Mutation detection by restriction enzyme site loss

The respective PCR products encompassing the target sequences were subjected to a restriction enzyme site loss assay. Briefly, genomic DNA was isolated from samples collected at 10, 15 and 21 dpi, depending on the experiment. The target region was PCR amplified using a specific primer set with Phusion high-fidelity polymerase (Supplementary Sequence 1). Purified PCR products (200 ng) were subjected to restriction enzyme protection analysis. The digested products were separated on a 2% agarose gel, cloned into the pJet2.1 vector, and subjected to Sanger sequencing to detect and characterize the indels.

### T7 Endonuclease I mutation detection assay

The T7EI assay was employed to measure mutations resulting from DSB repair through the NHEJ pathway, as described previously. Briefly, genomic DNA was prepared from samples collected at 10 and 15 dpi and used as a template for PCR amplification directly or after pretreatment with the respective restriction enzymes in order to enrich the NHEJ repair events. PCR amplicons of the target sequences (Supplementary Sequences 1) were denatured, renatured, and treated with T7EI. To calculate the frequency of modification, the PCR amplicons were cloned into the pJET2.1 cloning vector and sequenced by Sanger sequencing.

### Agroinfiltration

Binary constructs, including TRV RNA2 harboring the sgRNA, TRV RNA1, and TYLCSV, MeMV DNA-A, MeMV DNA-B, CLCuKoV, and CLCuMß infectious clones, were individually transformed into *Agrobacterium tumefaciens* strain GV3103 by electroporation. Transformed single colonies were grown overnight in selective medium, pelleted, and re-suspended. The bacterial cultures were mixed prior to infiltration and diluted to OD600 ~0.05 (for the infectious clones of DNA viruses) or 0.1 (for RNA1 and RNA2) in infiltration medium (10 mM MES [pH 5.7], 10 mM CaCl_2_, and 200 μM acetosyringone). The cultures were incubated at ambient temperature in the dark for 4–8 h. Mixed bacterial cultures were infiltrated into the 3–4-week-old leaves of *N. benthamiana* Cas9-OE plants using a 1 mL needleless syringe. Leaf disc samples were collected from inoculated plants, and systemic leaves at 15 and 21 dpi and were subjected to various molecular analyses to determine the presence of targeted modifications of the viral sequences.

### DNA blot analysis

PCR amplicons of IR-specific fragments of CLCuKoV and TYLCSV were DIG-labeled. Total genomic DNA (2 μg) from *N. benthamiana* plants was electrophoresed in 1% gels alongside the DIG-labeled size marker DIG marker-II (Roche). The DNA fragments were blotted onto a nylon membrane (Roche), followed by UV-crosslinking and hybridization with the respective IR-DIG-labeled probes. DNA bands were visualized using alkaline phosphatase-conjugated anti-DIG (1:10,000) and CPD chemiluminescent substrate in an Alpha Innotech digital imaging system.

### TRV-mediated delivery of sgRNA

TRV was used for the delivery and expression of sgRNAs with different target specificities as described previously^7^,^30^. To systemically deliver the sgRNAs, cloned TRV vectors were agroinfected into Cas9OE *N. benthamiana* plants for targeting of the TYLCSV, CLCuKoV, MeMV DNA-A, and MeMV DNA-B genomes, respectively. Seven days post TRV-mediated sgRNA delivery, a second leaf was infiltrated individually with agrobacterium containing infectious clones of TYLCSV, CLCuKoV with CLCuMß, and MeMV DNA-A with MeMV DNA-B. Fifteen days post-DNA viral challenge, systemic leaves were collected for molecular analysis.

### CRISPR/Cas9 evasion analysis of TYLCV2.3 and TYLCSV via sap transmission

TYLCV2.3 and TYLCSV sap inoculation was conducted as described^43^, with slight modifications. Briefly, two-week-old WT *N. benthamiana* plants were inoculated with sap extracted from TYLCSV-infected (CRISPR/Cas9 targeted) systemic leaves. The leaves were ground to a powder and re-suspended at 1:4 (w/v) dilution in potassium phosphate buffer (0.1 M, pH 8.0). The sap (from TYLCSV targeted at the IR or CP) was then applied to WT *N. benthamiana* plants. The adaxial surfaces of three leaves and pedicels were dusted with carborundum (200–450 mesh) and thoroughly rubbed with sap using a pestle. The leaf pedicels and main stem were also injected with the same sap using an 18-gauge needle. The top (young) systemic leaves were collected at 15 days after sap application and DNA was extracted. The extracted DNA was directly used for molecular analysis or enriched to detect the presence of indels by pretreatment with the respective enzymes. T7EI and restriction site loss were used for the detection of CRISPR/Cas9 evasion, and Sanger sequencing was used to identify the modified sequences of the viral escapees. These experiments were performed for the noncoding (IR) and coding (CP) viral regions.

### CRISPR/Cas9 evasion analysis of CLCuKoV via grafting

Scions from four-week-old WT *N. benthamiana* plants were grafted onto stocks of plants infected with CLCuKoV and CLCuMß and targeted at the IR and CP by CRISPR/Cas9 for 15 days. Top (young) leaves of scions were collected at 21 days after graft establishment and used for extraction of total genomic DNA. The extracted DNA was enriched for indels by pretreatment with the respective restriction enzymes, followed by use as a PCR template to amplify the IR or CP targets. Molecular analysis was performed for mutation detection as described above.

## Additional Information

**How to cite this article**: Ali, Z. *et al.* CRISPR/Cas9-Mediated Immunity to Geminiviruses: Differential Interference and Evasion. *Sci. Rep.* **6**, 26912; doi: 10.1038/srep26912 (2016).

## Supplementary Material

Supplementary Information
