# Corrigendum: CRISPR/Cas9-Mediated Immunity to Geminiviruses: Differential Interference and Evasion

**DOI:** 10.1038/srep30223

**Published:** 2016-08-26

**Authors:** Zahir Ali, Shakila Ali, Manal Tashkandi, Syed Shan-e-Ali Zaidi, Magdy M. Mahfouz

Scientific Reports 6: Article number: 2691210.1038/srep26912; published online: 05 26 2016; updated: 08 26 2016

This Article contains an error in Figure 5 where Figure 5D and 5E were published as Figure 5E and 5D respectively. The correct Figure 5 and correct figure legend appears below as Figure 1.

## Figures and Tables

**Figure 1:**
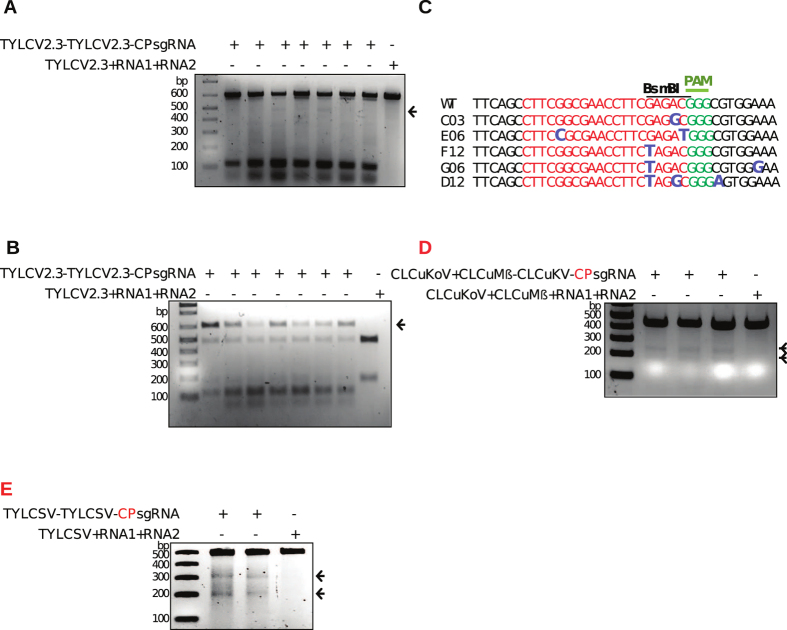
NHEJ-repaired CP sequence evades CRISPR/Cas9. **(A)** Evasion of repaired CP sequence of TYLCV genomes, as revealed by the T7EI assay. Sap from TYLCV-infected plants with an established CRISPR/Cas9 system against the CP region was applied to WT N. benthamiana plants. Total genomic DNA was isolated from top (young) leaves of sap-inoculated WT plants at 15 DAI. CP targets flanking PCR amplicons were subjected to T7EI. Arrows indicate the presence of the expected digested DNA fragment from samples of CP-targeted sap-infected plants compared to TRV empty vector with TYLCV. **(B)** BsmBI-recognition site loss assay for detecting escapees. BsmBI-treated PCR fragments were used as template in another round of PCR with the same primers. Purified DNA from this PCR was again subjected to BsmBI digestion. Arrowheads indicate the expected BsmBI-resistant DNA fragments compared to WT PCR amplicons from TRV empty vector. **(C)** Alignment of Sanger-sequencing reads of PCR amplicons encompassing the CP region of TYLCVfor mutation at the CRISPR/Cas9 targeting site. The wild-type (WT) TYLCV sequences are shown at the top (target sequence is shown in red, the BsmBI site by a line, and the protospacer-associated motif [PAM] is indicated in green; the various indels formed are shown in enlarged, bold, and blue font at their respective sites. **(D)** Evasion analysis of genomes of CLCuKoV genomes with repaired CP sequences via the T7EI assay. Wild-type scions were grafted to the stocks of CLCuKoV-infected plants with an established CRISPR/Cas9 system against the CP region. Total genomic DNA was isolated from top (young) leaves of WT scions at 21 DAI. **(E)** Evasion analysis of TYLCSV genomes with repaired CP sequences via the T7EI assay. For TYLCSV samples were prepared as in (A) CP targets flanking the PCR amplicons were subjected to T7EI. Arrows indicate the presence of expected digested DNA fragments from samples of CP-targeted sap-infected plants compared to TRV empty vector with TYLCSV.

