# Supplementary material for: CRISPR/Cas9-Mediated Immunity to Geminiviruses: Differential Interference and Evasion
Source: Sci Rep. 2016 May 26;6:26912. doi: 10.1038/srep26912 (PMC4881029; doi:10.1038/srep26912)
Supplement: Supplementary Information [file srep26912-s1.pdf]

SUPPLEMENTARY INFORMATION

CRISPR/Cas9-Mediated Immunity to Geminiviruses: Differential Interference and Evasion

Zahir Ali[1], Shakila Ali[1], Manal Tashkandi[1], Syed Shan-e-Ali Zaidi[1], and Magdy M. Mahfouz[1,*]

Laboratory for Genome Engineering, Division of Biological Sciences, 4700 King Abdullah University of Science and Technology, Thuwal 23955-6900, Saudi Arabia

*Correspondence: magdy.mahfouz@kaust.edu.sa

Table of Contents

Supplementary Figures.

Supplementary Figure 1. *SspI*-recognition site loss assay of CLCuKV targeted with variant IR-sgRNAs.

Supplementary Figure 2. Diagrammatic representation of genomes of different Geminiviruses.

Supplementary Figure 3. Long deletions at the IR region of MeMV.

Supplementary Figure 4. *SspI*-recognition site loss assay of MeMV targeted with variant IR-sgRNAs.

Supplementary Figure 5. *SspI*-recognition site loss assay for IR sequence modification of MeMV-B genome.

Supplementary Figure 6. Stem loop sequence in the IR of available geminiviruses.

Supplementary Figure 7. *SspI*-recognition site loss assay of TYLCSV targeted with variant IR-sgRNAs.

Supplementary Figure 8. Alignment of Sanger sequence reads for Indels in the CP and RCRII sequences of TYLCSV.

Supplementary Figure 9. Chromatogram of Sanger sequence reads for Indels in the IR of CLCuKV.

Supplementary Figure 10. Alignment of Sanger sequence reads for Indels in the IR of CLCuKV.

Supplementary Figure 11. Diagrammatic representation of the sap inoculation methods.

Supplementary Figure 12. *SspI*-recognition site loss assay of TYLCV2.3 targeted with IR-sgRNAs.

Supplementary Figure 13. Alignment of Sanger sequence reads for Indels in the CLCuKV escapees.

Supplementary Figure 14. Alignment of deduced amino acid sequences of CLCuKoV CP escapees.

Supplementary sequences and Maps

Supplemental sequence 1. TYLCV 2.3 genome sequence and map.

Supplemental sequence 2. TYLCSV genome sequence and map.

Supplemental sequence 3. CLCuKV genome sequence and map.

Supplemental sequence 4. CLCuMß genome sequence and map.

Supplemental sequence 5. MeMV-A genome sequence and map.

Supplemental sequence 6. MeMV-B genome sequence and map.

Supplemental sequence 7. IR-gRNAs sequences and maps.

Supplemental sequence 8. CP-gRNAs sequences and maps.

Supplemental sequence 9. RCRII-gRNAs sequences and maps.

Supplementary Tables

Supplementary Table 1. Primers used in this study.

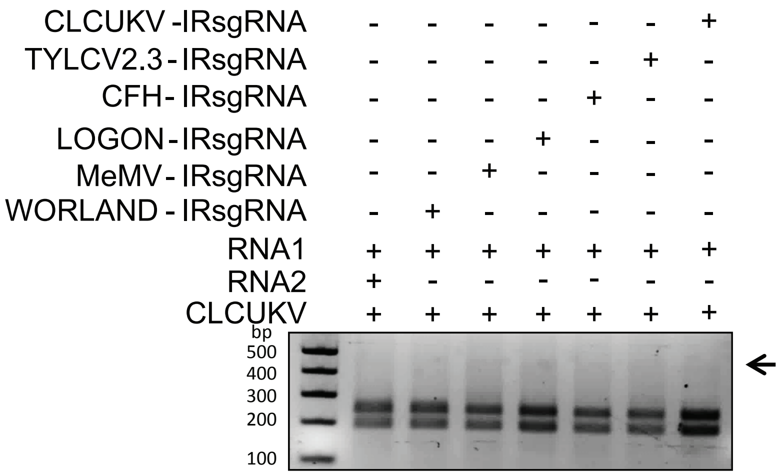

Supplementary Figure **1**. SspI-recognition site loss assay of CLCuKoV targeted with variant IR-sgRNAs. NHEJ repair analysis at the IR sequence of CLCuKoV. The CLCuKoV IR region (**446** bp) was analyzed for the loss of the SspI recognition site through NHEJ (Indels). Different variants of IR-sgRNAs were used to target IR of CLCuKV. Like the authentic CLCuKoV-IRsgRNA, none of the variant IRsgRNAs targeting of CLCuKoV IR sequence results in NHEJ repair. Arrow indicates the expected SspI resistant **446** bp bp DNA fragments. DNA fragments were resolved on **2%** agarose gel premixed with Ethidium Bromide stain.

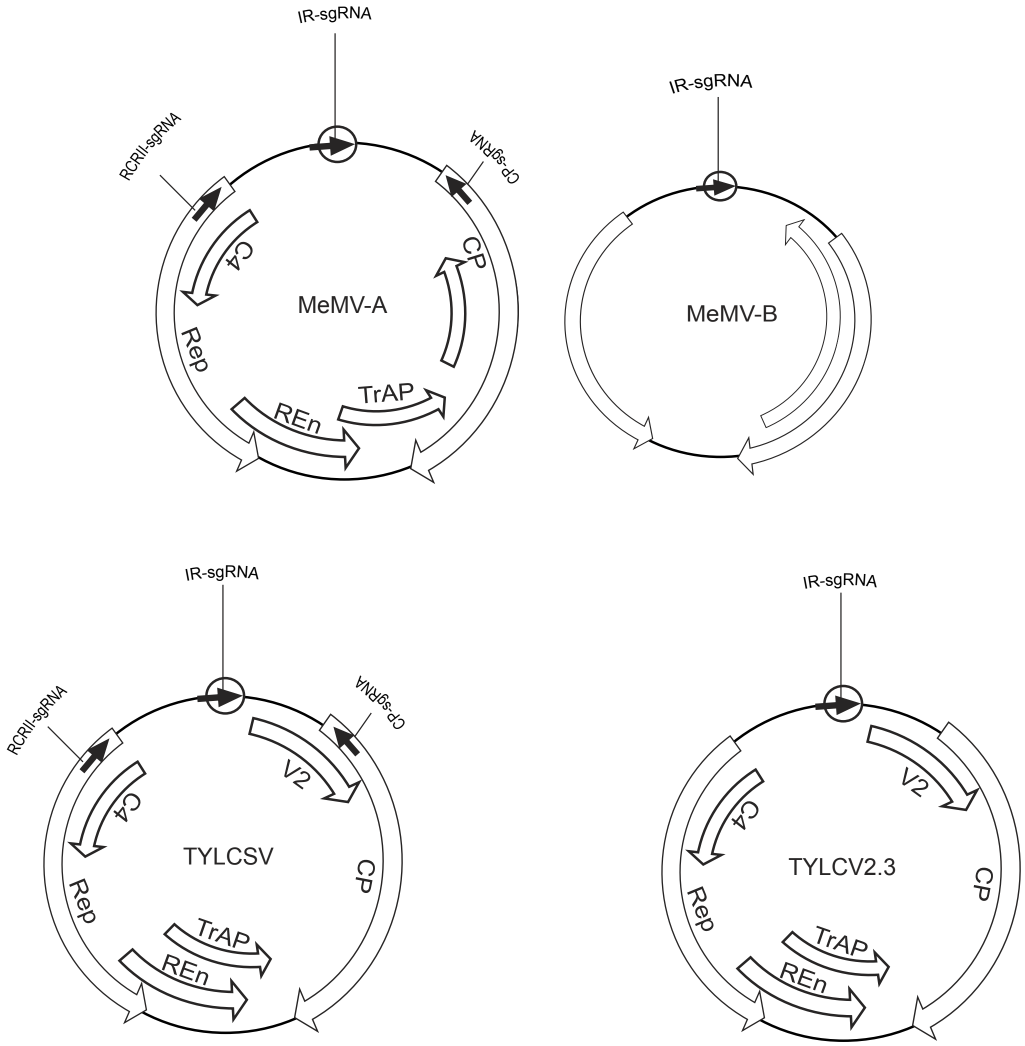

Supplementary Figure 2. Diagrammatic representation of genomes of different Geminiviruses.

Genome organization of the three selected viruses, bipartite (MeMV-A and MeMV-B), monopartite (TYLCSV and TYLCV2.3) are represented with targets (filled arrows, one in non-coding IR, one each in coding CP, or in Rep RCRII domain), IR is represented with open box and stem loop of nonanucleotide is represented by small circle, bidirectional ORFs (overlapping on inside) are represented by open arrows.

```
G12  CGGGGGAA--63Δ---ACCGGATG
H09  TCCCCCAA--112Δ--GATGGCCG
A06  TAAGGGAT--284Δ---CGTTTAA
```

Supplementary Figure 3. Long deletions at the IR region of MeMV.

Sanger sequencing of the IR of MeMV-A and MeMV-B, showing long deletions at the IR site. After targeting the IR region is PCR amplified and cloned into pJet2,1 and were sanger sequenced. Specific deletions are represented at their respective sites.

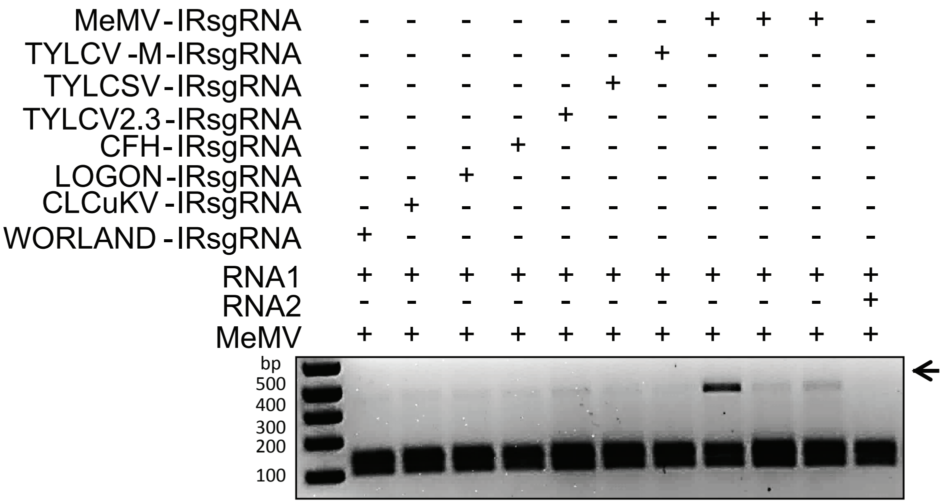

Supplementary Figure 4. ***SspI*-recognition site loss assay of MeMV targeted with variant IR-sgRNAs.**

IR sequence targeting and NHEJ repair in MeMV-A. The *SspI*-resistant IR fragment (446 bp) was analyzed for the loss of the *Ssp*I recognition. MeMV-A genome targeted (with different variants of IR-sgRNAs including the native MeMV-IRsgRNA) used was able to repair by NHEJ. Arrow indicating the expected *Ssp*I resistant 446 bp DNA fragments in all of the samples expressing an invariant IR-sgRNA. DNA fragments were resolved on 2% agarose gel premixed with Ethidium Bromide stain.

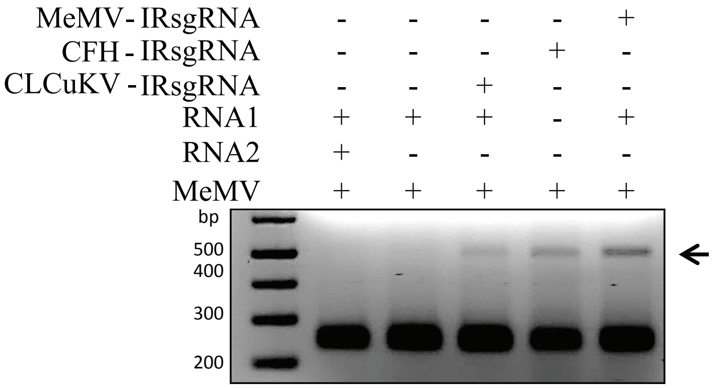

Supplementary Figure 5. ***SspI*-recognition site loss assay for IR sequence modification of MeMV-B genome.**

IR sequence targeting and NHEJ repair in MeMV-B. The *SspI*-resistant IR fragment (496 bp) was analyzed for the loss of the *Ssp*I recognition. MeMV-B genome targeted with MeMV-IRsgRNA used was able to repair by NHEJ. Arrow indicating the expected *Ssp*I resistant 496 bp DNA fragments in all of the samples compared to TRV alone. DNA fragments were resolved on 2% agarose gel premixed with Ethidium Bromide stain.

Supplementary Figure 6. Stem loop sequence in the IR of available geminiviruses.

The conserved nonanucleotide shown in Red, and flaking complementary sequence making the step loop of 143 Begomoviruses. Each sequence is represented with its accession number.

```
>AB055008
-GATAAAG-C-GGCCATCCG--TATAATATTACC--GGATGG-CCGCC--
>AB100304
--CAATAG-C-GGCCATCCG--TATAATATTACC--GGATGG-CCGCGA-
>AB100305
-CCAAAAG-C-GGCCATCCG--TATAATATTACC--GGATGG-CCGCG--
>AB162141
-CCAAAAG-C-GGCCATCCG--TATAATATTACC--GGATGG-CCGCG--
>AB267836
GGTTAAAG-C-GG-CACTCG--TATAATATTACC--GAGTG--CCGCGA-
>AB307731
-CCTAAAG-C-GGCCATCCG--TATAATATTACC--GGATGG-CCGCG--
>AB377111
---CCAAG-C-GGCC-CACGACTATAATATTACC----GTGGGCCGCGCA
>AB377113
---ACAAG-C-GGCC-CACGACTATAATATTACC----GTGGGCCGCGCA
>AB433979
--CCAAAG-C-GGCCATCCG--TTTAATATTACC--GGATGG-CCGCCG-
>AF012300
---CGTGG-C-GGCCATCCG-CTATAATATTACC--GGATGG-CCGCGC-
>AF039031
---CGTGG-C-GGCCATCCG-CTATAATATTACC--GGATGG-CCGCGC-
>AF084006
--CTCAAG-C-GGCCATCCG--TCTAATATTACC--GGATGG-CCGCGA-
>AF101476
---CGTGG-C-GGCCATCCG-ATATAATATTACC--GGATGG-CCGCGC-
>AF130415
---AAATT-C-GGCCATCCG-CAATAATATTACC--GGATGG-CCGCGA-
>AF188481
---AAAAG-C-GGCCATCCG-CACTAATATTACC--GGATGG-CCGCGA-
>AF189018
--ACGTGG-C-GGCCTGTAG--TATAATATTACC--TACAGG-CCGCCG-
>AF195782
--CTAAAG-C-GGCCATCCG--TATAATATTACC--GGATGG-CCGCGA-
>AF206674
--CAATCG-T-GGCCATCCG--TATAATATTACC--GGATGG-CCGCGA-
>AF261885
--CCAAAG-C-GGCCATCCG--ATTAATATTACC--GGATGG-CCGTGC-
>AF271234
--TTAAAG-C-GGCCATCCG--TATAATATTACC--GGATGG-CCGCGC-
>AF274349
--CAAAAG-C-GGCCATCCG--TCTAATATTACC--GGATGG-CCGCGC-
>AF291705
----GTGGCC-GGCCATCCG-ATATAATATTACC--GGATGG-CCGCGC-
>AF295401
```

--TAATTG-C-GGCCATCCG--TCTAATATTACC--GGATGG-CCGCGC-
>AF311734
--CAAAAG-C-GGCCATCCG--TATAATATTACC--GGATGG-CCGCGC-
>AF327436
--CAAAAG-C-GGCCATCCG--TATAATATTACC--GGATGG-CCGCGA-
>AF428255
--AAATTG-C-GGSCATCCG--TATAATATTACC--GGATGG-CCGCGC-
>AF490004
---CGTGG-C-GGCCATCCG-TTATAATATTACC--GGATGG-CCGCGG-
>AF511529
-TACAAAG-C-GGCC-CTCG--TATAATATTACC---GAGGG-CCGCGGG
>AJ132711
--AAAAAG-C-GGCCATCCG--TATAATATTACC--GGATGG-CCGCGC-
>AJ319674
--CAAAAG-C-GGCCATCCG--TATAATATTACC--GGATGG-CCGCGC-
>AJ319675
--CAAAAG-C-GGCCATCCG--TATAATATTACC--GGATGG-CCGCGA-
>AJ457985
--CAAAAG-C-GGCCATCCG--TATAATATTACC--GGATGG-CCGCGC-
>AJ457986
--CAAAAG-C-GGCCATCCG--TATAATATTACC--GGATGG-CCGCGA-
>AJ495812
--TAATCG-C-GGCCATCCG--TATAATATTACC--GGATGG-CCGCGA-
>AJ508784
---AATAT-C-GGCCATCCG-CAATAATATTACC--GGATGG-CCGCGA-
>AJ558116
--CAAAAG-C-GGCCATCCG--TATAATATTACC--GGATGG-CCGCGA-
>AJ558118
--CAAAAG-C-GGCCATCCG--TATAATATTACC--GGATGG-CCGCGA-
>AJ558119
--CAAAAG-C-GGCCATCCG--TATAATATTACC--GGATGG-CCGCGC-
>AJ558120
--TAAAAG-C-GGCCATCCG--TATAATATTACC--GGATGG-CCGCGA-
>AJ566744
--CAAAAG-C-GGCCATCCG--TATAATATTACC--GGATGG-CCGCGA-
>AJ608286
--ACGTGG-C-GGCCATCAG--TATAATATTACC--TGATGG-CCGCGC-
>AJ704603
--CAAAAG-C-GGCCATCCG--TATAATATTACC--GGATGG-CCGCGC-
>AJ865338
--TAAAAG-C-GGCCATCCG--TATAATATTACC--GGATGG-CCGCGC-
>AJ865339
--ATAAAG-C-GGCCATCCG--ATTAATATTACC--GGATGG-CCGCGC-
>AJ865340
--CCTTAG-C-GGCCATCCG--TATAATATTACC--GGATGG-CCGCGC-
>AM236784
--TAAAAG-C-GGCCATCCG--TATAATATTACC--GGATGG-CCGCGC-
>AM491778
--CAAAAG-C-GGCCATCCG--TATAATATTACC--GGATGG-CCGCGC-
>AM501481
--CTAAAG-C-GGCCATCCG--TTTAATATTACC--GGATGG-CCGCGC-
>AM701758

--TAAAAG-C-GGCCATCCG--ATTAATATTACC--GGATGG-CCGCGC-
>AM701761
--CAAAAG-C-GGCCATCCG--TATAATATTACC--GGATGG-CCGCGC-
>AM701765
--CCATAG-C-GCCCACCGT--TTTAATATTACC---GGTGG-GCGCGAA
>AM701768
--GTAAAG-C-GGCCATCCG--TCTAATATTACC--GGATGG-CCGCGC-
>AM884015
--CTATAG-C-GGCCATTCG--TATAATATTACC--GAATGG-CCGCGG-
>AM980509
--CAAAAG-C-GGCCATCCG--TATAATATTACC--GGATGG-CCGCGA-
>AY044137
--CTAAAG-C-GGCCATCCG--TATAATATTACC--GGATGG-CCGCGC-
>AY044139
--CCAAAG-C-GGTCATCCG--TATAATATTACC--GGATGG-CCGCGC-
>AY227892
--TTAAAG-C-GGCCATCCG--TATAATATTACC--GGATGG-CCGCGC-
>AY339618
--ACGTGG-C-GGCCATCCG--TTTAATATTACC--GGATGG-CCGCGC-
>AY456684
--TAATTG-C-GGCCATCCG--TCTAATATTACC--GGATGG-CCGCGC-
>AY502934
--TTAAAG-C-GGCCATCCG--TATAATATTACC--GGATGG-CCGCGC-
>AY508993
--ACGTGT-A-GGCCATCCG--TATAATATTACC--GGATGG-CCGCGC-
>AY514630
--CAATCG-T-GGCCATCCG--TATAATATTACC--GGATGG-CCGCGA-
>AY514632
--CAATCG-T-GGGCATCCG--TATAATATTACC--GGATGG-CCGCGA-
>AY602165
--GTAAAG-C-GGCCATCCG--TATAATATTACC--GGATGG-CCGCGA-
>AY754812
--AAAAAG-C-GGCCATCCG--TATAATATTACC--GGATGG-CCGCGA-
>AY754814
--TGATAG-C-GGCCATCCG--ATTAATATTACC--GGATGG-CCGCGG-
>AY927277
---CACAT-C-GGCCATCCG-CAATAATATTACC--GGATGG-CCGCGA-
>DQ116884
--AAAAAG-C-GGCCATCCG--TATAATATTACC--GGATGG-CCGCGC-
>DQ127170
--CAAAAG-C-GGCCATCCG--TATAATATTACC--GGATGG-CCGCGC-
>DQ207749
---CGTGG-C-GGCCATCCG-TTATAATATTACC--GGATGG-CCGCGC-
>DQ256460
--CAAAAG-C-GGCCATCCG--TATAATATTACC--GGATGG-CCGCGC-
>DQ336350
--CTATAG-C-GGCCATCCG--TATAATATTACC--GGATGG-CCGCCC-
>DQ339117
--AAAAAG-C-GGCCATCCG--TATAATATTACC--GGATGG-CCGCGA-
>DQ358913
--TTAAAG-C-GGCCATCCG--TCTAATATTACC--GGATGG-CCGCGC-
>DQ520943

```
----GTGG-C-GGCCATCCGTTTATAATATTACC--GGATGG-CCGCGC-
>DQ629101
--AAAAAG-C-GGCCATCCG--TATAATATTACC--GGATGG-CCGCGA-
>DQ629102
--AAAAAG-C-GGCCATCCG--TATAATATTACC--GGATGG-CCGCGA-
>DQ629103
----AAAG-CGGGCCATCCG-CACTAATATTACC--GGATGG-CCGCGA-
>DQ852623
--TAATTG-C-GGCCATCCG--TATAATATTACC--GGATGG-CCGCGC-
>DQ866128
--GTAAAG-C-GGCCATCCG--TTTAATATTACC--GGATGG-CCGCGA-
>DQ871221
--CAAAAG-C-GGCCATCCG--ATTAATATTACC--GGATGG-CCGCGA-
>EF011559
--CAAAAG-C-GGCCATCCG--TATAATATTACC--GGAGGA-CCGCGC-
>EF110891
--ATAAAG-C-GGCCATCCG--TATAATATTACC--GGATGGCCCGCG--
>EF194760
--TTAAAG-C-GGTCATCCG--TCTAATATTACC--GGATGG-CCGCGC-
>EF417915
---CGTGG-C-GGCCATCCG-TTATAATATTACC--GGATGG-CCGCGC-
>EU189149
--GTAAAG-C-GGCCATCCG--TATAATATTACC--GGATGG-CCGCGA-
>EU350585
--CCAAAG-C-GGCCATCCG--TATAATATTACC--GGATGG-CCGCGA-
>EU487025
-----ACG-CGTCCCACGTATAGTTAATATTACC----GTGGGACGCGCC
>EU487046
--CAAAAG-C-GGCCATCCG--TATAATATTACC--GGATGG-CCGCGA-
>EU596959
--CAATCG-C-GGCCATCCG--TTTAATATTACC--GGATGG-CCGCGA-
>EU624503
--GTAAAG-C-GGCCATCCG--TTTAATATTACC--GGATGG-CCGCGA-
>EU635776
--GTAAAG-C-GGCCATCCG--TATAATATTACC--GGATGG-CCGCGC-
>EU710749
--ACGTGG-C-GGCCATCCG--TATAATATTACC--GGATGG-CCGCGC-
>EU710752
-CACGTGG-C-GGCCATCCG-T--TAATATTACC--GGATGG-CCGCGA-
>EU710754
--CAAAAG-C-GGCCATCCA--TATAATATTACT--GGATGG-CCGCGC-
>EU862323
--CCAAAG-C-GGCCATCCG--TATAATATTACC--GGATGG-CCGCGA-
>EU910141
--CTAAAG-C-GGCCATCCG--TATAATATTACC--GGATGG-CCGCGA-
>FJ174698
---CGTGG-C-GGCCATCCG-CTATAATATTACC--GGATGG-CCGCGC-
>FJ237614
-CCAAAAG-C-GGCC-CTCG--TATAATATTACC---GAGGG-CCGCGAA
>FJ514798
--GCTAAA-C-GGCCATCCG--TATAATATTACC--GGATGG-CCGCGA-
>FJ956700
```

--AAAAAG-C-GGCCATCCG--TATAATATTACC--GGATGG-CCGCGC-
>FR873229
--GTAAAG-C-GGCCATCCG--TATAATATTACC--GGATGG-CCGCGA-
>GQ334472
--ACGTGG-C-GGCCATCCG--TATAATATTACC--GGATGG-CCGCGC-
>GU076442
--CTAAAG-C-GGCCATCCG--TATAATATTACC--GGATGG-CCGCGC-
>GU076454
--CTAAAG-C-GGCCATCCG--CATAATATTACC--GGATGG-CCGCGC-
>GU723730
--GTAAAG-C-GGCCATCCG--TTTAATATTACC--GGATGG-CCGCGG-
>GU732204
--AATCAG-C-GGCCATCCG--TATAATATTACC--GGATGG-CCGCGC-
>HF912280
--AAAAAG-C-GGCCATCCG--TATAATATTACC--GGATGG-CCGCGA-
>HM164541
--GTAAAG-C-GGCCATCCG--TATAATATTACC--GGATGG-CCGCCC-
>HM448898
--CTAAAG-C-GGCCATCCG--TCTAATATTACC--GGATGG-CCGCGC-
>HM461862
--CAAAAG-C-GGCCATCCG--TATAATATTACC--GGATGG-CCGCGC-
>HQ162270
--CAAAAG-C-GGCCATCCG--TATAATATTACC--GGATGG-CCGCGA-
>HQ201952
---CGTGG-C-GGCCATCCG-CTATAATATTACC--GGATGG-CCGCGC-
>JF803252
---CGTGG-C-GGCCATCCG-TTATAATATTACC--GGATGG-CCGCGA-
>JF803254
---CGTGG-C-GGCCATCCG-ATATAATATTACC--GGATGG-CCGCGC-
>JN381819
--CAAAAG-C-GGCCATCCA--TATAATATTACT--GGATGG-CCGCCC-
>JN564749
---CGTGG-C-GGCCATCCG-TTATAATATTACC--GGATGG-CCGCGC-
>JN680352
---AAATT-C-GGCCATCCG-CAATAATATTACC--GGATGG-CCGCGC-
>JQ714137
---CGTGG-C-GGCCATCCG-TTATAATATTACC--GGATGG-CCGCGC-
>JQ867093
--GTAAAG-C-GGCCATCCG--TTTAATATTACC--GGATGG-CCGCGA-
>JQ897969
--AAAAAG-C-GGCCATCCG--TATAATATTACC--GGATGG-CCGCGC-
>JX863081
-TTTCTAA---GGCCATCCG--TATAATATTACC--GGATGG-CCGCTC-
>JX972142
--CAAAAG-C-GGCCATCCG--TATAATATTACC--GGAAGG-CCCCCC-
>K02029
--ACGTGG-C-GGCCATCCG--TTTAATATTACC--GGATGG-CCGCGC-
>KC172826
--CAAAAG-C-GGCCATCCG--TATAATATTACC--GGATGG-CCGCGA-
>KC176780
--CAAAAG-C-GGCCATCCG--TATAATATTACC--GGATGG-CCGCGT-
>KC465466

```
--AAAAAG-C-GGCCATCCG--TATAATATTACC--GAATGG-CCGCGC-
>KC686705
--CCAAAG-C-GGCCATCCG--TATAATATTACC--GGATGG-CCGCGA-
>KC706615
--ACGTGG-C-GGCCATCCG--TATAATATTACC--GGATGG-CCGCGC-
>KC763630
--TTAAAG-C-GGCCATCCG--TATAATATTACC--GGATGG-CCGCGC-
>KC791690
---CGTGG-C-GGCCATCCG-TTATAATATTACC--GGATGG-CCGCGC-
>KC791691
-GCAAAAG-C-GGCCATCC---TATAATATTACCGGGGATGG-CCGC---
>KF150142
--GTAAAG-C-GGCCATCCG--AATAATATTACC--GGATGG-CCGCGA-
>KF551592
--CTAATC-C-GGCCATTCG--TATAATATTACC--GAATGG-CCGTGC-
>L14460
---CGTGG-C-GGCCATCCG-ATATAATATTACC--GGATGG-CCGCGC-
>S53251
--CTCAAG-C-GGCCATCCG--TCTAATATTACC--GGATGG-CCGCGA-
>X15656
--TTAAAG-C-GGCCATCCG--TATAATATTACC--GGATGG-CCGCGC-
>X61153
--GTAAAG-C-GGCCATCCG--TATAATATTACC--GGATGG-CCGCGC-
>X63015
--CAATCG-T-GGCCATCCG--TATAATATTACC--GGATGG-CCGCGA-
>X76319
---TAAAGCC-GGCCATCCG--TATAATATTACC--GGAGCT-CGGCGC-
>Y14874
--ACGTGG-C-GGCCATCCG--TATAATATTACC--GGATGG-CCGCGC-
>Y15034
---CGTGG-C-GGCCATCCG-CTATAATATTACC--GGATGG-CCGCGC-
>Z48182
--AAATTG-C-GGCCATCCG--TATAATATTACC--GGATGG-CCGCGC-
```

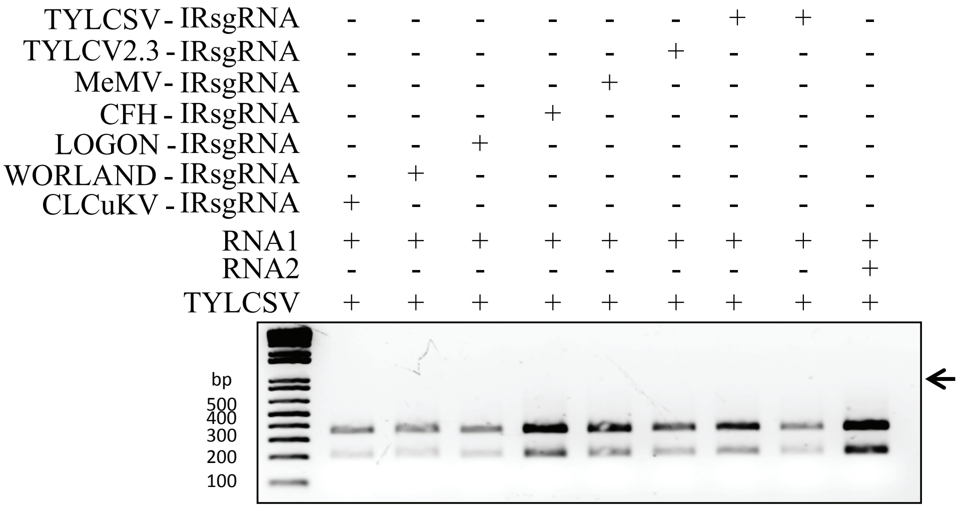

Supplementary Figure 7. *SspI*-recognition site loss assay of TYLCSV targeted with variant IR-sgRNAs.

NHEJ repair analysis at the IR sequence of TYLCSV. The TYLCSV IR region (562 bp) was analyzed for the loss of the *Ssp*I recognition site through NHEJ (Indels). Different variants of IR-sgRNAs were used to target IR of TYLCSV. Like TYLCSV-IRsgRNA, none of the variant IRsgRNAs targeting of CLCuKV IR sequence results in NHEJ repair. Arrow indicating the expected *Ssp*I resistant 562 bp DNA fragments. DNA fragments were resolved on 2% agarose gel premixed with Ethidium Bromide stain.

A

```
                                              PAM
WT   ACGAGACCGAAGGCCGTATGGAACTA-GTCCTA-TGGATTTTGGTC
B01  ACGAGACCGAAGGCCG--------------CTA-TGGATTTTGGTC -13
C02  ACGAGACCGAAGGCCGTATGGA--------CTA-TGGATTTTGGTC -07
E02  ACGAGACCGAAGGCCGTATGGAACTA--------TGGATTTTGGTC -06
H05  ACGAGACCGAAGGCCGTATGGAACTAA---CTA-TGGATTTTGGTC -03+1
C01  ACGAGACCGAAGGCCGTATGGAACTA-GTCCTAATGGATTTTGGTC +01
```

B

```
                                              PAM
WT   TTTACCCTCGAACTGAATGAGCATGTGGAG-ATGAGGTTGCCCATCT
G07  TTTACCCTCGAACTGAATGAG----------ATGAGGTTGCCCATCT -09
B11  TTTACCCTCGAACTGAATGAGCA--------ATGAGGTTGCCCATCT -07
A08  TTTACCCTCGAACTGAATGAGCATGTGGAGGATGAGGTTGCCCATCT +01
E11  TTTACCCTCGAACTGAATGAGCATGTGGAGAATGAGGTTGCCCATCT +01
```

Supplementary Figure 8. Alignment of Sanger sequence reads for Indels in the CP and RCRII sequences of TYLCSV.

Alignment of Sanger sequence reads of PCR amplicons encompassing the CP (A) and RCRII (B) target for Indels sequence confirmation. The wild-type (WT) sequences are shown at the top (target sequence is shown in red; the protospacer-associated motif [PAM] is indicated by green, followed by the various indels formed, indicated by numbers to the right of the sequence [-, deletion of x nucleotides; +, insertion of x nucleotides; and >, change of x nucleotides to y nucleotides].

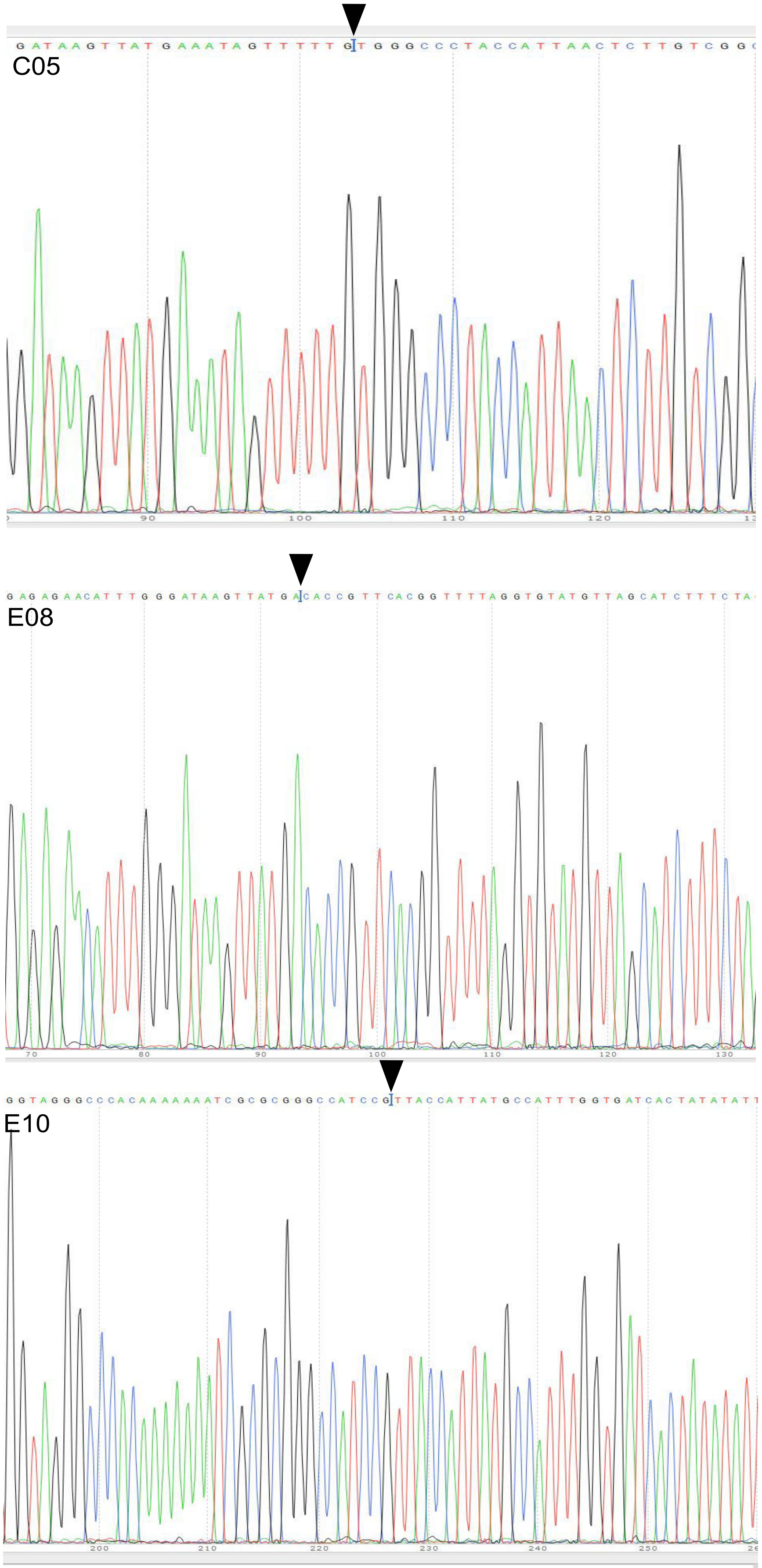

Supplementary Figure 9. Chromatogram of Sanger sequence reads for Indels in the IR of CLCuKV.

Sanger-sequencing chromatograms of PCR amplicons of IR region of CLCuKV targeted with IR-sgRNA. PCR amplicons show the respective sequence long deletions after targeting by CRISPR/Cas9.

```
G04  ACCGATTGACC--128Δ---TACCGGATGG
F11  TCGGTGATCAC--199Δ---TCGTTGCTAA
F03  TGTCGGCCAATC--62Δ---CTTGTCGGCC
```

Supplementary Figure 10. Alignment of Sanger sequence reads for Indels in the IR of CLCuKoV.

Alignment of Sanger-sequencing reads of PCR amplicons of IR region of CLCuKV targeted with an invariant IR-sgRNA. Total DNA was extracted and the PCR amplicons were cloned and sanger sequenced. PCR amplicons show the respective sequence mutation at CRISPR/Cas9 targeting site.

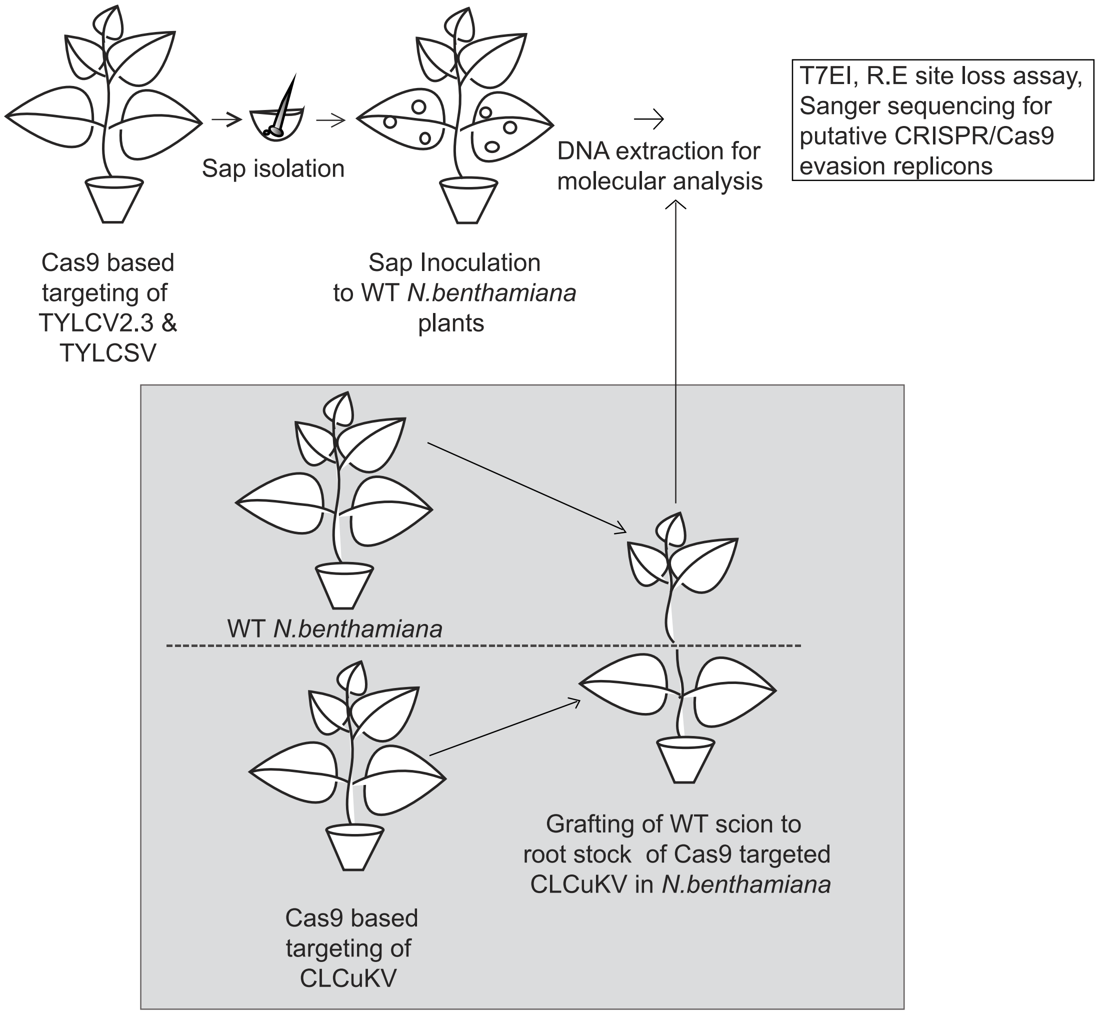

Supplementary Figure 11. Diagrammatic representation of the sap inoculation methods.

Sap was isolated from plants in which TYLCV2.3 was targeted with CRISPR/Cas9 at CP or IR. The extracted sap was rub inoculated with Carborundum, injected to the leaf pedicels and main stem directly. Alternatively for CLCuKoV (in gray box), a scion from the wild *N benthamiana* plant was grafted to a CRISPR/Cas9 targeted CLCuKoV plants. Collected samples were used for different molecular assays.

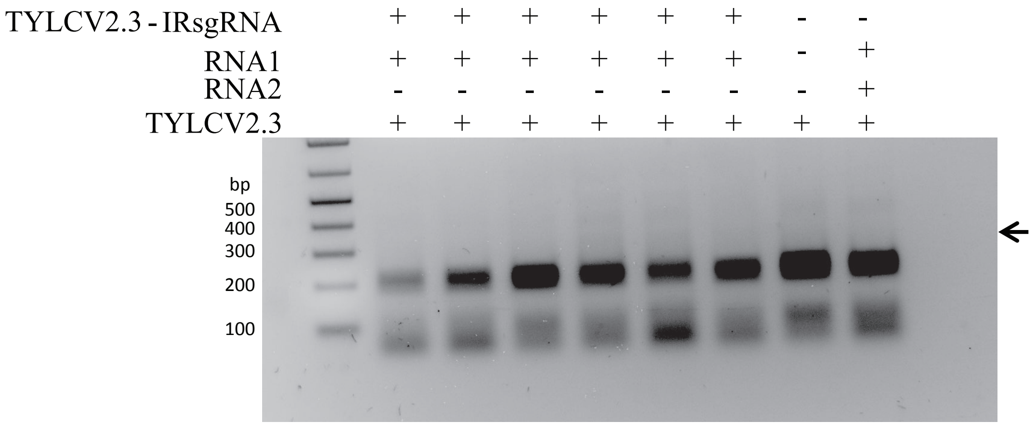

Supplementary Figure 12. ***SspI*****-recognition site loss assay of TYLCV2.3 targeted with IR-sgRNAs.**

NHEJ repair analysis at the IR sequence of TYLCV2.3. The IR region was analyzed for the loss of the *Ssp*I recognition site through NHEJ (Indels). None of sample show any SspI resistant DNA fragment. Arrow indicates the expected *Ssp*I resistant DNA fragments. DNA fragments were resolved on 2% agarose gel premixed with Ethidium Bromide stain.

```
                                                     PAM
WT   AGCAGCACGGCTCACATATGGGCTGTCGAAGTTG-AGACGGCGGCGTACCTTCGAA
ESC1 AGCAGCACGGCTCACATATGGGCTGTCGAA---G-AGACGGCGGCGTACCTTCGAA -03
ESC2 AGCAGCACGGCTCACATATGGGCTGTGGAA---G-AGACGGCGGCGTACCTTCGAA -03,C>G
ESC3 AGCAGCACGGCTCACATATGGGCTGTGAAAGTTG-AGACGGCGGCGTACCTTCGAA -G>A
ESC4 AGCAGCACGGCTCACATATGGGCTGTGGAAGTTGCAGACGGCGGCGTACCTTCGAA +01
```

Supplementary Figure 13. Alignment of Sanger sequence reads for Indels in the CLCuKoV escapees.

Alignment of Sanger-sequencing reads of PCR amplicons of CP region of CLCuKV. Total DNA was extracted from the WT scion grafted to virus (CLCuKV and CLCuMß) inoculated and CRISPR/Cas9 machinery expressing plants. Reads of the PCR amplicons show the respective sequence mutation at CRISPR/Cas9 targeting site. The wild-type (WT) sequences is shown at the top (target sequence is shown in red; and the protospacer-associated motif [PAM] is indicated by green, the various indels formed are shown enlarge, bold and in blue at their respective sites.

A

WT	Ile	Ser	Thr	**Pro**	**Val**	**Ser**	**lys**	**Val**	**Val**	**Arg**	**Arg**	**Arg**	Leu	Asn	Phe	Asp
C03	Ile	Ser	Thr	**Pro**	**Ala**	**Ser**	**lys**	**Val**	**Val**	**Arg**	Arg	Arg	Leu	Asn	Phe	Asp
E06	Ile	Ser	Thr	**Pro**	**Ile**	**Ser**	**lys**	**Val**	**Val**	**Arg**	Gly	Arg	Leu	Asn	Phe	Asp
F12	Ile	Ser	Thr	**Pro**	**Val**	*******	**lys**	**Val**	**Val**	**Arg**	Arg	Arg	Leu	Asn	Phe	Asp
G06	Ile	Ser	Thr	**Pro**	**Val**	*******	**lys**	**Val**	**Val**	**Arg**	**Arg**	**Arg**	Leu	Asn	Phe	Asp
D12	Ile	Ser	Thr	**Pro**	**Ala**	*******	**lys**	**Val**	**Val**	**Arg**	Arg	Arg	Leu	Asn	Phe	Asp

B

WT	Val	Arg	Arg	Arg	**Leu**	**Asn**	**Phe**	**Asp**	**Ser**	**Pro**	**Tyr**	Val	Ser	Arg	Ala
ESC1	Val	Arg	Arg	Arg	**Leu**	**---**	**Phe**	**Asp**	**Ser**	**Pro**	**Tyr**	Val	Ser	Arg	Ala
ESC2	Val	Arg	Arg	Arg	**Leu**	**---**	**Phe**	**His**	**Ser**	**Pro**	**Tyr**	Val	Ser	Arg	Ala
ESC3	Val	Arg	Arg	Arg	**Leu**	**Asn**	**Phe**	**Asp**	**Ser**	**Pro**	**Tyr**	Val	Ser	Arg	Ala
ESC4	Val	Arg	Arg	Arg	**Gln**	**Leu**	*******	**Gln**	**Pro**	**Ile**	**Cys**	Glu	Pro	Cys	Cys

Supplementary Figure 14. Alignment of deduced amino acid sequences of CLCuKoV CP escapees.

Amino acid sequence alignment of CP region of CLCuKoV. Targeted CP region Sanger sequencing reads were converted to amino acid sequence. A) TYLCV2.3, B) CLCuKoV. Missense mutations are shown in blue, deletions with blue dots, nonsense with asterisks and frame shift resulting in premature stop with green. The wild-type (WT) amino acids sequences is shown at the top (amino acids in the target sequence are shown bold and in bigger font)

Supplementary Sequence 1; *Cotton Leaf Curl Kokhran virus* (CLCuKV; AJ496286)

ACCGGATGGCCGCGCGATTTTTTTGTGGGCCCTACCATTAACTCTTGTCGGCCAATCATATGACGCGCTCAAAGCTT
AAATAATTCTCCCGCTTATTATAAGTACTTCGTTGCTAAGTATGCGTTTGAAAAATGTGGGATCCACTGTTAAATGA
GTTCCCCGACACCGTTCACGGTTTTAGGTGTATGTTAGCAGTTAAATATTTGCAGTTAGTACAGAAAACTTACTCTC
CTGATACATTGGGTTACGATTTGATAAGGGATTTAATCCTGGTAATAAGGGCTAGGAATTATGTCGAAGCGACCA
GCAGATATAATCATTTCCACGCCCGCTTCGAAGGTACGCCGCCGTCTCAACTTCGACAGCCCATATGTGAGCCGTG
CTGCTGCCCCCATTGTCCGCGTCACCAAAGCAAAAGCATGGGCGAACAGGCCCATGAACAGAAAGCCCAGGATGT
ACAGGATGTACAGAAGTCCAGATGTTCCTAGAGGATGTGAAGGTCCATGTAAGGTTCAGTCGTTTGAGTCCAGAC
ATGATATTCAGCATATAGGTAAAGTAATGTGTGTTAGTGATGTTACTCGTGGTACTGGGCTGACCCATAGAGTTGG
TAAGAGATTTTGTGTTAAGTCTGTTTATGTGTTGGGTAAGATCTGGATGGATGAGAACATTAAGACGAAGAATCAC
ACGAATAGTGTGATGTTTTTCTTGGTTAGAGATCGTAGACCTGTTGATAAACCTCAAGATTTTGGAGAGGTATTTA
ATATGTTTGATAATGAGCCCAGTACGGCGACTGTGAAGAATGTTCATCGTGATAGGTATCAAGTTCTGCGCAAATG
GTATGCAACTGTCACCGGTGGACAATACGCTTCAAAGGAACAAGCTCTCGTGAAGAAATTTATTAGAGTTAATAAT
TATGTTGTGTATAACCAGCAGGAAGCTGGCAAGTATGAGAATCATTCTGAGAATGCTTTAATGTTGTATATGGCGT
GTACTCACGCCTCTAACCCAGTGTATGCTACCTTGAAGATACGGATCTACTTCTATGATTCCGTGACAAATTAATAG
ATATTGAATTTTATTGAAGATGATTGGTCTACAAATACAACATGTTGTAATACATTCCATAATACATGATCAACTGC
TCTAACTACATTATTAATACTGACAATTCCTAAGTTATTTAAATATTTAAGCACTTGAGTCCTAAAGACCCTTAAGAA
ACGACCAGTCGGAGGCTGTGAGGTCATCCAGATTCGGAAAGCTATGAAACATTTGTGTATCCCCAACGCTTTCCTC
AGGTTGTGATTGAACTGTATCTGGACGGTGATGATGTCTCTGTTCATTAGGAATGCTCGATTGTGGTGCTCTGTTA
TCTTGAAATACAGGGGATTTTGAATCTCCCAGATAAACACGCCATTCTCTGCTTGAGCTGCAGTGATGAGTTCCCCT
GTGCGTGAATCCATGGTTGTGGCAGGCTAATGCTATGAAGTACGAACACCCACAAGGGAGATCAACTCTCCGACG
TCTGGTCCCCTTCTTGGCTAGCCTGTGCTGCACTTTGATTGGAACCTGAGTAGAGTGGGCTCTCGAGGGTGATGAA
GATTGCATTCTTTAAAGCCCAATTTTTGAGTGCAGAATTCTTCTCTTCATCCAAAAACTCTTTATAGCTTGAATTGGG
TCCTGGATTGCAGAGGAAGATAGTGGGAATTCCGCCTTTAATTTGAACTGGCTTCCCGTATTTTGTATTTGATTGCC
AGTCCTTTTGGGCCCCCATGAACTCCTTAAAGTGCTTTAGGTAATGCGGGTCGACGTCATCAATGACGTTAAACCA
GGCGTCATTACTGTATACCCTTGGGCTCAGATCTAGATGTCCACACAGATAATTATGTGGACCTAATGATCTGGCC
CACATCGTCTTCCCCGTCCTACTGTCACCCTCAATCACTACACTTATTGGTCTATTGGCCCGCGCAGCGGCACTGAC
GACGTTCTCGGCAGCCCACACTTCAAGTTCTTCTGGAACTTGATCGAAAGAAGAAGAGGAAAAAGGAGAAACATA
AGGAGCTGGTGGCTCCTGAAAGATTCTGTCTAGATTTGCATTTAAATTATGAAATTGCAGTACAAAATCCTTAGGA
GCTAGTTCCTTAATGACTCTAAGAGCCTCCGACTTACTTCCCGCGTTAAGTGCTGCGGCGTAAGCGTCATTGGCTGT
CTGTTGCCCTCCTCTTGCTGACCTTCCGTCGATCTGAAATTGCCCCCAGTCGAGAATGTCCCCGTCCTTCTCGATGTA
GGATTTGACGTCGGAGCTGGATTTAGCTCCCTGTATGTTCGGATGGAAATGTGCTGACCTGCTTGGGGAGACCAA
GTCGAAGAATCGCATATTCTGGCACTTGAATTTCCCTTCGAACTGGATGAGAACATGCAAGTGAGGAGTCCCATCT
TCGTGAAGCTCTCTGCAGATTCTAATATATTTTTTTGAAGTTGGGGTTTGTATATTTAATAATTGGGAAAGTGCTTC
CTCTTTGGTGAGAGAACATTTGGGATAAGTTATGAAATAGTTTTTGGAATAAATACCGTTCCGCTTTGGAGGCATG
TTGACTAAAATTGAATCACCGATTGACCGTTCTTGCAAACTCTCCCCGGTATATCGGTGATCAATATATAGTGATCA
CCAAATGGCATAATGGTAATAAAAAAACTTTAATTTGAAATTCAAACCAAAAGGCTAAAGCGGCCATCCGTTTAAT
ATT

Target sequences are shown in gray.

Cotton leaf curl Multan Betasatellite (CLCuMB; AJ298903)
accgtgggcgagcggtgtccgatggtttcttggtgggtcccattgctggtattgacttgatttgacttatattgggccaatttaatgggttgaaaatgtttg
ggcctttggaagagggtcttttatggaatagggctctgtatattgttggtgatatgtgtgttaaatatgcattgctggtttgtgtttggaatttaaacggtg
aactttttattgaatacgtacggttcgtttacatccattcccaatatctctgggttttcaagtacaagtatatcaagtctgtgaactatatcttctatctcga
tctcttctatctttgccccgttgtatgcgaataggaaattcgctatgatgctcccttcaaagccgttgaagtcgaatggaacgtgtatgtcttcgtacgtgt
actggacgatcccttcatacttgattagcgatggtgactttgtggatagtatcctcatgtgaatgaagatcttcatattctccatgatgcgaacgtcgact
atgaacctgactccctccttgtttgttccgctcggtgtcatttctgcttatttgatggaaatgtttagtgtgcatgcatttatagacttaatgaacagatattt
tgtgtagttgtgtggttgtgagtgatttattattatgtgattgtccattaaagggataaagtgatgatggagacgtattacacgtgttgtcatgttggcttg
aaatctttatacatgggtttgtgccggtatacctatatatacggatagaaaacggatgagaaaaggaaaaacggaactgaagaggaaaaacaaaga
aaagaaacaaggatatattatttatgaaagaaatgggagcgcagcgaatcgaaacaggaaaacccaaggaaagagaaaaaattaaaagtaaaga
gaaaaaaaattcaaattcgaaaacgtcatcgtttgagagtaataaaaaaaaagaaaaaacaaaaacatatccgaaaacgttgtcgtttgaaggtgt
ctgtgtggttttaccatttactgtgtggtaaatggtaagtgattgaagaatagttaaaaaatggaggcccgataggtaaattgtaccccaatatattggg
gttcaattggggactcataaattgcctttcctaaaatacccccgcttttgtgtctaagaggcgcgtcggagtgcgcctataaagttaacattctctctcct
cttttgatctccaatacaatttcccggtgatcggagtcgaattttccgacacgcgcggcggtgtgtacccctgggagggtaggtaccactacgctacgca
gcagccttagctacgccggagcttagctcgcccacgttctaatatt

Supplementary Sequence 2;

Merremia Mosaic Virus (MeMV-A)

ACCGGATGGCCGCCCGCCGCGCCCCCCCTGGGCCCACATATTAAAGCCGTCCAATCACAAAGCGTCCTGGAAGTCT
AATTGTTTAAAATAAGCCTATAAATACATTGGAGTCCGTCTATACCCCACCAACTTTAATTTAAAATGGTTAAGAGG
GACGCCCCATGGCGTTTAATGGCGGGGACCACTAAAGTTAGTCGCAACGCCAATTTCTCGCCACGTGGAGGTATG
GGCCCTAAGGCCGCTGCTTGGGTTAACAGGCCCATGTACAGGAAGCCCAGAATTTATCGCACTTTGAGAGGGCCT
GATGTTCCTAAAGGTTGTGAAGGCCCATGTAAGGTACAGTCTTTCGAGCAGCGTCATGATATTTCTCATGTTGGTA
AGGTAATCTGTATATCCGATGTAACTCGTGGTAACGGTATTACTCACCGTGTTGGCAAGCGTTTTTGTGTGAAGTCT
GTGTATATTCTAGGTAAAATATGGATGGATGAGAACATAAAGCTGAAGAACCACACGAACAGCGTCATGTTTTGG
TTGATTCGTGACAGGAGACCCTATGGTACCCCTATGGATTTTGGTCAGGTGTTTAACATGTATGACAATGAGCCGA
GTACTGCTACCGTCAAGAACGATCTTCGCGATCGATTTCAAGTCATGCATAGGTTCTATGCCAAAGTAACTGGTGG
TCAGTATGCCAGTAACGAGCAGGCATTGGTTCGGCGATTTTGGAAGGTTAACAACTACGTCGTGTATAACCATCAG
GAAGCAGGAAAATACGAGAATCACACGGAGAATGCTCTGTTATTGTATATGGCATGTACTCATGCTTCTAATCCTG
TGTATGCTACCTTGAAAATTCGTAGTTATTTTTATGACTCCATTTCGAATTAATAAAGATTAAATTTTATTGAATGTC
TTTCGAGCACACAATTTACATATGGTTTATCCGTTGCGAAACGAACAGCTCTAATGACATTGTTAAGCGAAACAAC
ACCTAATTGATCTAAATACATTAAAACTAAATCTTTAAATCTATTTAAATATGTCGTCCCAGAAGCTTGAACTGATGT
CGTCCAGATTTGGAAGTTCAGGTATGCTTTGTGTAGACTCAACGCCTTCCTCAGGTTGTAGTTGAACCGTATTTGGA
TGGTGTATATTCTGGTTGTGGTGTATATTGGCTCCTCCACTTGGATTATCTTGAAATAGAGGGGATTTGGAACCTCC
CAGATAAAAACGCCATTCATTGCTTGATGAGCAGTGATGGGTTCCCCTGTGCGTGAATCCATGGTTTCTGCAGTTG
ATGTGTACGTAAATTGAACAGCCACAGTCCAGGTCTAACCTTCTCCGTCTAATGACAGATTTGGATCTCTTCGCTCT
CCTGTGCTGTGCTTTGATAGAGGGGGGAGTTGAGGAAGATGAATTTTGCATTGTGGATCGTCCACGCTCTGAGAG
ATGCGTTTTCATCTTTATCGAGGAAGTCTTTATAGCTAGCCCCCTCTCCTGGATTGCACAGCACGATTGAGGGTATT
CCTCCTTTAATTTGAACTGGCTTCCCGTATTTACAGTTGGACTGCCAGTCCTTTTGGGCCCCTATCAATTCTTTCCAA
TGCTTTAATTTCAAATAATTAGGGCTTATGTCATCAATGACGTTATATTCGACGTCATTCGAATAGACTCTGCCATTA
AAGTCAAGATGTCCACTAAGATAATTATGTTTACCTAATGAACGGGCCCACATTGTTTTTCCAGTTCGACTATCTCC

TTCGATGATGATACTTATCGGTCTATCTGCCCGCGCAGCGGCACCCCTCCCAAAGTAGTTGTCTGCCCATTCTTGCA
TCTCATCTGGAACGTTCGTGAAGGAGGAGAGTTGAAACGGAGGAACCCATGGTTCTGGAGACTTCTGAAATATTC
TTGTTGCGTTCGCAACGAGATTGTGATGTTGAAGAAAGAAGTGTTGTGGTTGTTCCTCCTTTATTATTTGCAGTGCT
TCCTCTGCAGAAGCTGCGTTTAACGCCTTTGCGTATGTATCGTTAGAAGACTGCTGACCTCCTCTAGCAGATCTTCC
GTCGATCTGGAATTGTCCCCATTCAATTGTATCTCCGTCCTTGTCGATGTAGGACTTGACATCGGAGCTGGATTTAG
CTCCCTGAATGTTCGGATGGAAATGTGCTGACCTGGTTGGGGATACCAAATCGAAGAATCTGTTATTCGTGCAGTT
GTATTTTCCTTCGAACTGGATAAGCACATGGAGATGAGGTTCCCCATTATCGTGAAGCTCTCTACAGATCTTGATGA
ATTTTTTGTTTACAGGGGTGTGCAGAGCTTTGATTTGGGACAGTGCTTCTTCTTTGGCTAATGAACATATAGGGTAT
GTGAGGAAATAGTTTTTGGCTTTTATTGAGAATGAACCCTTCCGTGGCATTTTTGTAATAAGGGATGTTCCCCCAAT
TGCTCCGCTCTCAAAACTCTATATGAATCGGGGGAACTGGGGGTACATTTATACTAGAACTCTCATTAAAGGGATT
TGCAACACGTGGCGGCCATCCGCTATAATATT

Target sequences are shown in gray.

Merremia Mosaic Virus (MeMV-B)

ACCGGATGGCCGCCCGCCGCGCCCCCCCTGGCCCGCCCACCGCGCCCCTTGACCCCACATGGTGCCCACCAGCTGT
GATGCACtTTGTAAAGGTAATACGTGGAACGCTtGTATGCATTCATTTGAACTAACTGTTAACTTTCTTCTTTAATTT
GAATTATTGTCGCGCTACTATGAAAGGTTGGGTCATACTTACAATGATTAAATTGTTTAACGTAATGTACGATTTGG
AGACGTGGACCAGTTATAACCATTATACATCGTCAAATTAGTTTGCCGTCTCTTTAAGATCTATATATATATATGCTT
GAACGTGGAGTTATGTAAACATATATTTTATTAATATCTATTACTAGAATATGTATTCTTTTAGATATAGACCGTATC
ATTTTAATTATCGGAAACGATTTTACACACGTACGCAAGTGTCTAAACGGGTAGCCCCGTTTAAACGAGCTGATGT
GAAATGTCGAACGAGGCAGACGACTATCGTTCATGATGAGACTAAGATGTCTTCGCAGCGCATTCATGAGAACCA
ATTTGGTCCAGAGTTTGTGATGACACATAACTCCGCCATATCCACTTTCATTAATTATCCTACTTTGAGTAAGACCG
AGCCTAACAGAAGCAGATCTTACATTAAGTTGAAACGCTTGCGGTTTAAGGGAACTGTTAAAATTGAGCGTGTGT
ATGCGGATATGAACATGGATGGTTTGAACCCTAAAGTTGAAGGGGTATTCACTCTTGCTGTTGTAGTTGACCGAAA
ACCTCATTTAAAGCCATCTGGATGTCTGCATACATTTGATGAGGTATTTGGTGCACGGATTCACAGTCATGGTACGT
TAGCCATTACTCCGTCACTGACAGATCGTTACTACATTCGCCATGTGTTTAAACGTGTAATGTCTGTTGAGAAGGAT
ACTGCCATGGTTGATGTGGAAGGATCGATGTCTCTCTCTAATAAGCGTTTTAATTGTTGGGCTACGTTTAAGGATCT
TGATCATGAATCTTGTAAGGGTGTTTATGACAATATTAGCAAAAACGCCTTGTTAATTTATTATTGTTGGATGTCTG
ATGTACCATCTAAGGCATCGTCATTTGTATCATTTGATTTGGATTATGTTGGCTAAATAAAAATATGTAATTTCTGTT
TTAAATAAATAACTGTGTATTGAACAATAAATTCTATTTTAACGATTTCGGCTGAGCCGGTGTACAATTACTATTAA
TACACTCATGGACCGTTGATCGTACAAGATCATTTAATTGGGCCATTGACATTGTTATATTGGATTGGGCCCTTGAA
GCCCCTACGATTGAAGCAGAATCACCTGGATCCAACGCACTGGTTCCCAGACGATTGAGTTGCCTGTATGGGTGTA
TTGCATTTTCCAGTTCCGAATCTGTGTCCGTTTGGCCTATACCGATTGTGCTTCTTGAGGCCCATGACTCGCCTGGT
AATAATTCTATTGGGCTTGGTAGCCCAATTCTTGCCATTGAGATTGACCGGATCATTTTTCTTTCCCATCTCCCGTAG
CCCACATGTGAGAAATCGACATCCTTCTCGGAAAATTGCTTAGACAGGATCTTCACCGCCGGAGCTCTGAATGGGA
TATCTACAGAATGTTTCGCTGTCGACAGTTTTAGTTTCCCTTTGAACTTGGCGAAATGTGTTCGTTGGTGTACGTTT
GAATCACAGACTCTGTAGTATAGTTTCCACGGGATTGGGTCTTTCAGTGAGAAGAAAGACGAGGAAAAGTAATGA
AGGTCTATGTTGCATCTGATTGGGAACGTCCATGATGCTTGTAATGATTCGTTATCCGTCATCCTTCTGTCGTGGAT
CTCCACAATTACAGTACCTGTTGCGTTTATCGGTACCTGTTGCCTGTATTCTATGACGCAGTGATCTATTTTCATACA
ACTGCGACTTAATCTTGCTGATAATTGAGCCGCTGCTGACGGAAAGTGCAGTATTATCTCAGTTAAATCATGAGAT
AGCTGATATTCGTCTCTGTGAGATTCTATATAATTAAAGGCACTTGGAGGATTAACCAATTGAGCATCCATATAGC

AAAATTAGCCCGCGCAGCGGAATGGATCTAACTGAGAGACGTCGTGGTGATTAATATAGGATGTTTTCTTGAAGA
AAAGAAGATGATATTTGGGAGAGTAGATCTGGATGTTTGTGAGGTTTTTGTTGTGAAGAAGTATATGTGGTTCTGT
CTATATATAGACTTTATTAATGTATTGGAAATAAACTAATAACAATTGCTTCATTGACACGTTTATGGTTGTACTATG
TCTCTTACTGTTGCTAAAGAAGTTTAAGATCTATACTGGAAGCACTTAGTGGCATTTGTGTAATAAGGGATGTTCCC
CCAATTGCTAAGGGgTGTTCCCCAATTGCTCCGCTCTCAAAACTCTCTATGAATTGGGGGAACTGGGGGTACATTT
ATACTAGAACTCTCATTAAAGGGATTTGCAACACGTGGCGGCCATCCGCTATAATATT

Target sequences are shown in gray.

Tomato yellow leaf curl Sardinia virus; (TYLCSV; L27708)

TAATATTACCGGATGGCCGCGCTCCCCGATAAAGTAGTGGGCCCTACGCACTAATTTGTGTCGACCAATGAAAATG
CAGCCTCAAAGCTTAAATAATGTATTTACTTTGTTATAAACTTGGCTTCTAAGTTTTGAAAAAACCATCAATATGTG
GGATCCTCTATTAAATGAATTTCCAGATTCAGTTCATGGTCTCCGTTGTATGCTTGCAATAAAATATTTGCAGCTAG
TTGAAGAAACCTATGAACCCAATACTCTGGGTCACGATCTAATTAGGGATCTCATTTCCGTCATTCGTGCTCGTGAC
TATGCCGAAGCGAACAGGCGATATACTAATGTCAAGCCCGCTCTCGAAGTTTCGTCGAAAACTGAACTTCGACAGC
CCGTATACCAGCCGTGCTGCTGCCCCCACTGTCCAAGGCATCAAGCGTCGATCATGGACTTACAGGCCCATGTATC
GAAAGCCGCGGATGTACAGAATGTACAGAAGCCCTGATGTCCCGTTTGGTTGTGAAGGTCCTTGTAAAGTCCAGT
CGTATGAGCAGCGTGACGACGTCAAGCATACCGGTGTTGTTCGTTGTGTTAGTGATGTAACTAGGGGTTCTGGTAT
TACACATAGAGTAGGTAAACGGTTTTGTATTAAGTCAATCTATATTTTAGGGAAGATTTGGATGGATGAAAATATA
AAAAAACAAAATCATACTAACCAGGTCATGTTCTTTTTAGTACGAGACCGAAGGCCGTATGGAACTAGTCCTATGG
ATTTTGGTCAAGTTTTTAACATGTTTGATAATGAACCTAGTACGGCTACTGTGAAGAACGATTTAAGGGATAGGTA
CCAAGTAATGAGGAAGTTCCATGCCACGGTGGTAGGTGGTCCGTCAGGGATGAAGGAGCAGTGTCTGTTGAAGA
GGTTTTTTAAAGTTAATACCCATGTAGTTTATAATCATCAAGAGCAGGCGAAGTATGAAAACCATACTGAGAATGC
GTTGTTGTTGTATATGGCATGTACTCATGCTTCTAACCCAGTGTATGCTACGTTGAAAATACGTATCTATTTTTATGA
TGCTGTAACAAATTAATAAAGATTGTATTTTATTTCATGTTGTTCAATTACATCTATTGTGTTCTCAAATACATCGAA
TAAGACATAATCAACTGCTCTAATAACATTGTTTAATGAAATTACACCTATATTATTCAAATACTTATACACTTGAAA
CCTAAAGACTCTTAAAAAATGACCAGTCGGAGACTGTAAGGTCGTCCAGATGCGGAAGTTGAGAAAACATTTGTG
AATCCCCAGTGCCTTCCTCAAGTTGTGGTTGAACCTGATTTGAAATGTTAGTAAATTGTATTTGCTGTTGAATGGTT
GTTGGTTGTGTCTGGTTATCGTGAAATATAGGGGATTTGTTATCTCGAAGGTATAAACGCCACTCGTTGCTTGATG
CGCAGTGATGTACTCCCCTGTGCGTAAATCCATGGTTTATGCAGTCTAAATGTATATAGTATGAACAGCCACAGTC
CAGATCTACCCTTCTACGCCTCACTTGTCTCTTCTTGGCGATGTGGTGTTGGATCTTGATTGGTATTTGTGAACAAT
GGCTGGTGGATGGTGACGAAGATTGCATTTTTTATGGCCCAATTTTTTAATGTTTGATTTTTTTCTTCGTCGAGGTA
TTCTTTAAATGATGATTGTGGGCCTGGATTGCAGAGGAAGATAGTGGGTATGCCTCCTTTAATTTGAATGGGCTTC
CCATACTTTGTGTTGCTTTGCCAGTCCCTTTGGGACCCCATGAATTCTTTAAAGTGTTTTAAATAATGCGGGTCTAC
GTCATCAATGACGTTGTACCAAGCATTATTGCTATATACTTTTTGACTGAGGTCAAGATGTCCGCACAAATAATTAT
GTGGGCCTAGGGAACGGGCCCACATAGTCTTTCCTGTCCGGCTGTCACCCTCAATCACTATACTCACCGGCCTCCA
AGGCCGCGCAGCGACATCCATGACGTTCTCGGAAACCCAGTGTTCAAGTTCATCCGGAACTTGATCAAAAGAAGA
AGATAAAAAAGGAGAAACATATGGTGCCGGAGGCACCTGAAAAACCATATTTAAATTACTATTTATATTATGAAAA
TGTAAAATGTAATCTCTAGGGGCTAATTCTTTAATTACATCAAGAGCCTCCGACTTACGTCCTGCGTTAATTGCCTT
GGCGTAAGCGTCATTGGCTGTCTGCTGTCCTCCCCTTGCAGATCGTCCGTCGATCTGGAAAGTACCCCATTCAAGA
ACATCTCCGTCCTTGTCGATGTAGGACTTGACGTCGGAGCTTGATTTAGCTCCCTGAATGTTCGGATGGAAATGTG

CTGATCTGGTTGGGGATACCAGATCGAAGAATCTGTTATTTTTGCAGTTGAATTTACCCTCGAACTGAATGAGCAT
GTGGAGATGAGGTTGCCCATCTTCGTGTAATTCTCTGCAAATCTTAATGTATTTTTTATTTGTTGGTGTTTGTAGTTG
AAGTAGTTGTTCTAGGGCTTCTTCTTTGGAGAGAGAACATTTTGGGAAAGTTAGAAAATAATGTTTTGCATTTATTT
GAAAACGCTTAGGCTGAGCCATTTGGTCAATGGGTACCAATTGACCTCAGATTCATTTTATTCCATGTATTGGTAGA
TTGGTAGCTCTTATATACCTGGGTACTAAATGGCATGTATGTAAATATGTTAAGTTATTTTTTTAATTTATTTTAATTT
TTGGAATTTTAGCGGCCATCCGTT

Target sequences are shown in gray.

Tomato yellow leaf curl virus (TYLCV2.3)

ACCGGATGGCCGCGAATTTTGTGTGGGCCCCTCAACGCACTAACTGACAAGGACATGCGAACCAATCAAATTGCA
TCCTCAAACGTTAGATAAGTGTTCATTTGTcTTTATATACTTGGTCCCCAAGTATTTTGTCTTGCAATATGTGGGACC
CACTTCTAAATGAATTTCCTGAATCTGTTCACGGATTTCGTTGTATGTTAGCTATTAAATATTTGCAGGCTGTTGAG
GAAACTTACGAGCCCAATACATTGGGCCACGATTTAATTAGGGATCTTATATCTGTTGTAAGGGCCCGTGACTATG
TCGAAGCGACCAGGCGATATAATCATTTCCACGCCCGTCTCGAAGGTTCGCCGAAGGCTGAACTTCGACAGCCCAT
ACAGCAGCCGTGCTGCTGTCCCCATTGTCCAAGGCACAAACAAGCGACGATCATGGACGTACAGGCCCATGTACC
GAAAGCCCAGAATATACAGAATGTATCGAAGCCCTGATGTTCCCCGTGGATGTGAAGGCCCATGTAAAGTCCAGT
CTTATGAGCAACGGGATGATATTAAGCATACTGGTATTGTTCGTTGTGTTAGTGATGTTACTCGTGGATCTGGAAT
TACTCACAGAGTGGGTAAGAGGTTCTGTGTTAAATCGATATATTTTTTAGGTAAAGTCTGGATGGATGAAAATATC
AAGAAGCAGAATCACACTAATCAGGTCATGTTCTTCTTGGTCCGTGATAGAAGGCCTTATGGAAGCAGCCCAATG
GATTTTGGACAGGTTTTTAATATGTTCGATAATGAGCCCAGTACCGCAACCGTGAAGAATGATTTGCGGGATAGGT
TTCAAGTGATAAGGAAATTTCATGCTACAGTTATTGGTGGGCCCTCTGGAATGAAGGAACAGGCATTAGTTAAGA
GATTTTTTAGAATTAACAGTCATGTAACTTATAATCATCAGGAGGCAGCCAAGTATGAGAACCATACTGAGAACGC
CTTGTTATTGTATATGGCATGTACGCATGCCTCTAATCCAGTGTATGCAACTATGAAAATACGCATCTATTTCTATG
ATTCAATATCAAATTAATAAATTTTATATTTTATATCATGACTTTCTGTTACATTTATTGTGTTTTCAAGTACATCATA
CAATACATGATCAACTGCTCTGATTACATTGTTAATGGAAATTACACCAAGACTATCTAAATACTTAAGAACTTGAT
ATCTAAATACTCTTAAGAAACGACCAGTCTGAGGCCGTAAGGTCGTCCAGATTTGGAAGTTGAGATAACATTTGTG
AATCCCCAGTACCTTCCTGATATTGTGATTGAATCTTATCTGTATTGAAATGATGTCGTGGCTCATTAGAAATGGCC
TCTCGTCGTGGTTGGTGATCTTGAAATATAGGGGATTTTCTATCTCCCATATAAAAACGCCATTCTGGGCTTGATGA
GCAGTGATGAGTTCCCCGGTGCGTGAATCCATGATTGATGCAGTTGATGTGGAGGTAATATGAGCATCCGCAGTC
GAGGTCTATGCGCTTACGTCTGACTGGCTTAGTCTTCGCTATGCGGTGTTGGATTTTGATTGGCACTTGAGAACAG
TGGCTCGTAGAGGGTGACGAAGGTTGCATTCTTGAGAGCCCAATTTTTCAAGGATATGTTTTTTTCTTCGTCTAGAT
ATTCCCTATATGATGAGGTAGGTCCTGGATTGCAGAGGAAGATAGTGGGAATTCCCCCTTTAATTTGAATCGGCTT
CCCGTACTTTGTGTTGCTTTGCCAGTCCCTCTGGGCCCCCATGAATTCCTTGAAGTGCTTTAAATAATGCGGGTctac
gtcatcgatgacgttgtaccacgcatcattactgtacacctttggacttaggtctagatgtccacataaataattatgtgggcctagagacctggcccac
attgtcttccccgttctgctatcaccctcgatgacaatactattaggtctCCATGGCCGCGCAGCGGAAGACATGACGTTCTCGGACA
CCCATACTTCAAGTTCATCTGGAACTTGATTAAAAGATGAAGATAAAAAGGGAGAAATATAAGGAGCCGGAGGCT
CCTGAAAAATTCTATCTAAATTTGAATTTAAATTATGAAATTGAAGTATAAAGTCTCTAGGAGCTTTCTCCTTCAGTA
TATTGAGGGCCTGAGCTTTGGACCCTGAATTGATTGCCTCGGCATATGCGTCGTTGGCAGATTGGCAACCTCCTCT
AGCTGATCGTCCATCGACTTGGAAAACTCCATGATCAATGACGTCTCCGTCTTTTTCCATATAGGATTTGACATCGC
TTGAACTCTTAGCTCCCTGAATGTTCGGATGGAAATGTGCTGACCTGGTTGGGGATGTGAGGTCGAAGAATCTGTT

GTTTTTGCACTGGAACTTTCCTTCGAACTGGATGAGCACATGCAAGTGAGGAGTCCCATCTTCATGAAGCTCTCTGC
AGATTCTAATGAATTTTTTGGAAGTGGGTGTTTGTATATTTAATAATTGGGAAAGTGCTTCCTCTTTAGTTAGAGAG
CATTTGGGATAAGTGAGAAAATAATTTTTGGCATTTATTTTAAACCGATTGGGGGCTGCCATATTGACTTGGTCAAT
CGGAGTCTCTCAACTCTTTCTATGTATTGGTGTATTGGAGTCCTATATATATGGAGACTCCAATGGCATATATGTAA
ATATTGTACTTTAATTCAAAATCATCACGCTCCAAAAAGCGGCCATCCGTATAATATT

Target sequences are shown in gray.

Supplementary Table 1. Primers used in this study.

primers name	sequence (5' ---- 3')	Usage
TYLCV2.3-IR-T-F	AATTGGGAAAGTGCTTCCTCT	TYLCV IR flanking region
TYLCV2.3-IR-T-R	ATAGTCACGGGCCCTTACAACA	
TYLCV-IR-T1	CGAGTCTAGAGGCCATCCGTATAATATTA CGTTTTAGAGCTAGAAATAGCAAG	To clone TYLCV IR-sgRNA
SPDK-gRNA-R	acatGCCCGGgAAAAAAAGCACCGACTCGG	To clone all sgRNA
TYLCSV-IR-F	CGAACTGAATGAGCATGTGG	To amplify IR region for T7EI and SspI assay And probe synthesis
TYLCSV-IR-R	TGCAAGCATACAACGGAGAC	
TYLCSV-IR-T CLCUKV-IR-T	CGAGTCTAGAGGCCATCCGTTTAATATTA CGTTTTAGAGCTAGAAATAGCAAG	To clone TYLCSV and CLCuKoV IR-sgRNA
TYLCSV-RCRII-T	CGAGTCTAGAGTGAATGAGCATGTGGAG ATGGTTTTAGAGCTAGAAATAGCAAG	To clone TYLCSV-RCRII-sgRNA
TYLCSV-RCRII-R	CATGCCATTTAGTACCCAGGT	To amplify RCRII region for T7EI and RSL assay
TYLCSV-RCRII-F	TCCTGCGTTAATTGCCTTGG	
TYLCSV-CP-F	AAGCCGCGGATGTACAGAAT	To amplify CP region for T7EI and RSL assay
TYLCSV-CP-R	ACTTCGCCTGCTCTTGATGA	
TYLCSV-CP-T	CGAGTCTAGAGCCGTATGGAACTAGTCCT AGTTTTAGAGCTAGAAATAGCAAG	To clone TYLCSV-CP-sgRNA
CLCUKV-CP-F	GTACTTCGTTGCTAAGTATGCGTTT	To amplify CP region for T7EI and RSL assay
CLCUKV-CP-R	TATGCTGAATATCATGTCTGGACTC	
CLCUKV-CP-T	CGAGTCTAGAGATGGGCTGTCGAAGTTGA GAGTTTTAGAGCTAGAAATAGCAAG	To clone CLCuKoV-CP-sgRNA
CLCUKV-RCRII-F	GACCTTCCGTCGATCTGAAAT	To amplify RCRII region for T7EI and RSL assay
CLCUKV-RCRII-F	AGAGTTTGCAAGAACGGTCAAT	
CLCUKV-RCRII-T	CGAGTCTAGAGCTGGATGAGAACATGCA AGTGGTTTTAGAGCTAGAAATAGCAAG	To clone CLCuKoV-RCRII-sgRNA
MeMV-IR-T-F	TGTGCAGAGCTTTGATTTGG	To amplify IR region for T7EI and RSL assay
MeMV-IR-T-R	AAATTGGCGTTGCGACTAAC	
MeMV-IR-T	CGAGTCTAGAGCCATCCGCTATAATATTACGTT TTAGAGCTAGAAATAGCAAG	To clone MeMV-IR-sgRNA
MeMV-RCRII-T	CGAGTCTAGAGTGGATAAGCACATGGAGATG GTTTTAGAGCTAGAAATAGCAAG	To clone MeMV-RCRII-sgRNA
MeMV-RCRII-T-F	CGTTTAACGCCTTTGCGTAT	To amplify RCRII region for T7EI and RSL assay
MeMV-RCRII-T-R	AGAGTTTTGAGAGCGGAGCA	
MeMV-CP-T	CGAGTCTAGAGACGTGGAGGTATGGGCCCTA GTTTTAGAGCTAGAAATAGCAAG	To clone MeMV-CP-sgRNA
MeMV-CP-T-F	AAAGCCGTCCAATCACAAAG	To amplify CP region for T7EI and RSL assay
MeMV-CP-T-R	TCCTGTCACGAATCAACCAA	
MeMVB-IR-F	GCTTCATTGACACGTTTATGGTT	To amplify IR region of MeMV for T7EI and RSL assay
MeMVB-IR-R	TGGTTATAACTGGTCCACGTCTC	
